# Incorporation of Manganese Complexes within Hybrid Resol-Silica and Carbon-Silica Nanoparticles

**DOI:** 10.3390/nano11030774

**Published:** 2021-03-18

**Authors:** François-Xavier Turquet, Montserrat Corbella, Clémentine Fellah, Gilles Montagnac, Bruno Reynard, Laurent Bonneviot, Kun Zhang, Belén Albela

**Affiliations:** 1 Laboratoire de Chimie, Ecole Normale Supérieure de Lyon, Université de Lyon, 46 Allée d’Italie, CEDEX 07, 69364 Lyon, France; fx.turquet@protonmail.com (F.-X.T.); laurent.bonneviot@ens-lyon.fr (L.B.); 2 Departament de Química Inorgànica i Orgànica (Secció Inorgànica), Universitat de Barcelona, Martí I Franquès 1-11, 08028 Barcelona, Spain; 3 Laboratoire de Géologie, Ecole Normale Supérieure de Lyon, Université de Lyon, 46 Allée d’Italie, CEDEX 07, 69364 Lyon, France; clementine.fellah@ens-lyon.fr (C.F.); gilles.montagnac@ens-lyon.fr (G.M.); bruno.reynard@ens-lyon.fr (B.R.); 4 Shanghai Key Laboratory of Green Chemistry and Chemical Processes, Department of Chemistry, East China Normal University, 3663 North Zhongshan Rd., Shanghai 200062, China; kzhang@chem.ecnu.edu.cn

**Keywords:** hybrid materials, manganese complexes, mesoporous silica, nanoparticles, resol, carbon, anthracene carboxylate, luminescent properties

## Abstract

The incorporation of a luminescent probe into a nano-vector is one of the approaches used to design chemosensors and nanocargos for drug delivery and theranostics. The location of the nano-vector can be followed using fluorescence spectroscopy together with the change of environment that affects the fluorescence properties. The ligand 9-anthracene carboxylate is proposed in this study as a luminescent probe to locate two types of manganese complexes inside three series of porous nanoparticles of different composition: resol-silica, carbon-silica and pure silica. The manganese complexes are a tetranuclear Mn^III^ cluster [Mn^III^_4_(μ-O)_2_(μ-AntCO_2_)_6_(bpy)_2_(ClO_4_)_2_] with a butterfly core, and a Mn^II^ dinuclear complex [{Mn^II^(bpy)(AntCO_2_)}_2_(μ-AntCO_2_)_2_(μ-OH_2_)]. The magnetic measurements indicate that both complexes are present as dinuclear entities when incorporated inside the particles. Both the Mn complexes and the nanoparticles are luminescent. However, when the metal complexes are introduced into the nanoparticles, the luminescent properties of both are altered. The study of the fluorescence of the nanoparticles’ suspensions and of the supernatants shows that Mn^II^ compounds seem to be more retained inside the particles than Mn^III^ compounds. The resol-silica nanoparticles with Mn^II^ complexes inside is the material that presents the lowest complex leaching in ethanol.

## 1. Introduction

Nanomaterials possessing multiple functionalities is a blooming field of science boosted by the search of novel tools for nanomedecine, sensor technology, optical devices, nanochemistry where interfaces and hybrid precursors are at stake. Shape, composition and porosity may be suitably combined with specific electrical, magnetic or luminescent properties. In the last decades, different multifunctional materials have been developed, showing applications in fields such as optoelectronics, drug delivery and green chemical processes. All of these are key issues in our society. In most of the cases, serendipitous methods were used. They lacked molecular control at the interfaces. Therefore, it was difficult to optimize the final features of the material. Recent studies using more rational designs of multifunctional materials propose new strategies. One of them is the use of phosphorescent inorganic nanoparticles such as quantum dots that can be applied in optical biosensing and imaging [1]. An alternative strategy is to incorporate a luminescent probe in the nanoparticle, which is one of the approaches used in the field of chemosensors and porous nanocargos for drug delivery and theranostics [2,3,4,5]. For example, a recent study has reported a photoactive manganese(I) complex for CO delivery with a fluorescent ligand (dansylimidazole) [6]. This complex is luminescent and exhibits CO release under low-power visible light. Due to its luminescent properties, the cellular internalization of the pro-drug molecules can be visualized by fluorescence spectroscopy in real time.

The aim of using a luminescent ligand is that, first, the position of the system can be determined through fluorescence microscopy. And second, that modification of the environment of the fluorophore (complexation, contact with the matrix or the solvent) would quench, amplify or otherwise modify the luminescence of the ligand, thus providing information on its behaviour in or outside the matrix (nanoparticle, micelle, polymeric vesicle, etc). In this work, we propose to study a multifunctional system based on porous nanoparticles of different composition. To this we incorporate a manganese complex. This compound possesses a luminescent ligand derived from anthracene (9-anthracene carboxylate), which provides an excellent opportunity either to track it in a biological medium, if the final application of the material is drug delivery, or to develop building blocks for multifunctional nanodevices.

The anthracene emission is measured in the 400–500 nm range and is widely reported in the literature [7]. It presents two sets of absorption bands, from 220 to 280 nm, and 290 to 400 nm. They correspond to π → π* transitions and have a distinctive vibronic structure due to transition moments oriented through different molecular axes. The position of these bands, their quantum yield and lifetime are independent of the solvent polarity [8,9,10,11]. However, this is not the case for the 9-anthracene carboxylic acid, which displays sensitivity to the polarity of its environment [12,13,14]. Modification of the luminescence properties of the 9-anthracene carboxylic acid has been widely discussed, and multiple explanations are proposed: acid-base equilibrium, solvent or concentration-dependent formation of dimers or higher order aggregates, and structural reorganisation of the anthracene ring occurring in excited states [11,15,16,17,18,19,20,21,22]. Thus, the modification of the 9-anthracene carboxylate ligand luminescence can provide information on its coordination, concentration, and the pH of its environment. 

Herein we present the synthesis of three series of porous nanoparticles with silica, resol-silica and carbon-silica composition, and the incorporation of Mn^II^ and Mn^III^ complexes possessing the luminescent ligand 9-anthracene carboxylate into these nanoparticles. The resulting materials are characterized using a panel of physico-chemical techniques, and a focus on the discussion of the luminescent properties is presented. 

## 2. Materials and Methods

### 2.1. Synthesis of the Nanoparticles

The three types of nanoparticles were synthesized according to the method reported by Zhang and coworkers [23]. All the reactants and solvents were used as received without any further purification. The two manganese complexes, [Mn^III^_4_(μ-O)_2_(μ-AntCO_2_)_6_(bpy)_2_(ClO_4_)_2_] and [{Mn^II^(bpy)(AntCO_2_)}_2_(μ-AntCO_2_)_2_(μ-OH_2_)], with AntCO_2_ = 9-anthracene carboxylate, were synthesized as described elsewhere [24]. They are called [Mn^III^] and [Mn^II^], respectively, in the following sections.

#### 2.1.1. Mesoporous Resol-Silica Nanoparticles (RSNP)

Mesoporous resol-silica nanoparticles (RSNP) were synthesized by using a one-pot soft templating method [23]. Resorcinol (5.0 g, 4.5 × 10^−2^ mol) and cetyltrimethylammonium bromide (CTAB, 5.0 g, 1.4 × 10^−2^ mol) were mixed together in an aqueous solution (EtOH 200 mL/water 500 mL) of triethanolamine (TEA, 10.6 g, 7.1 × 10^−2^ mol). The solution was then stirred (140 rpm) for 30 min at room temperature. At this point, pH was 9.0. A solution of formaldehyde was then added (5.0 mL, 37 wt%), followed, two minutes later, by the incorporation of tetraethoxysilane (TEOS, 25 mL, 1.1 × 10^−1^ mol). The pH then increased to 9.1. The reaction mixture was first stirred for 24 h at room temperature, then heated up to 80 °C for another 24 h (final pH = 8.0). The suspension of aggregated particles was then filtered and the solid washed a couple of times with a solution of HCl in ethanol (2.0 mol/L), then with pure ethanol. The material was subsequently dried in an oven overnight at 80 °C, yielding 16 g of product. Thermogravimetrical analysis (TGA) showed a ratio of 43% silica and 55% resorcinol (the missing 2% comes from water absorbed within the porosity and silica condensation at high temperature). IR spectrum (cm^−1^): 3424 (bd), 2929 (w), 2855 (w), 1624 (s), 1506 (w), 1474 (w), 1448 (w), 1384 (w), 1152 (bd), 1085 (s), 969 (m), 799 (m), 556 (w), 462 (s). 

#### 2.1.2. Mesoporous Carbon-Silica Nanoparticles (CSNP)

Thermal treatment of RSNP in a tubular furnace under a gentle N_2_ flux led to the carbonisation of the polymer phase. Temperature was increased at the rate of 3 °C/min up to 900 °C, and maintained in these conditions for 3 h. Residual mass was 61% of the initial sample. TGA analysis showed a ratio of 64% silica and 33% carbon. IR spectrum (cm^−1^): 3449 (bd), 1608 (bd), 1384 (w), 1152 (bd), 1090 (s), 965 (w), 818 (m), 467 (s).

#### 2.1.3. Mesoporous Silica Nanoparticles (SNP)

Biphasic RSNP were calcined in a tubular furnace under a gentle air flux in order to remove the organic polymer. Temperature was increased at the rate of 3 °C/min up to 550 °C, and maintained in these conditions for 6 h. Residual mass at 1100 °C (silica) was 46% of the initial sample. IR spectrum (cm^−1^): 3445 (bd), 1645 (s), 1389 (w), 1152 (bd), 1088 (s), 968 (m), 800 (m), 565 (w), 464 (s).

### 2.2. Synthesis of [Mn]@NP

#### 2.2.1. Synthesis of Mesoporous [Mn^III^]@Resol-Silica Nanoparticles ([Mn^III^]@RSNP)

[Mn^III^]@silica-resol mesoporous nanoparticles were synthesised by impregnation of the desired amount of [Mn^III^] compound (10, 30 and 50 mg for materials 1-[Mn^III^]@RSNP, 3-[Mn^III^]@RSNP and 5-[Mn^III^]@RSNP, respectively), which corresponded to around 1, 3 and 5 wt% of [Mn^III^] complex, respectively) with 1.00 g of RSNP in acetonitrile (200 mL). The dispersion was stirred at room temperature for 24 h before filtration of the solid. The powder was then dried overnight in an oven at 80 °C. Anal. Found (wt%): 1-[Mn^III^]@RSNP, C, 27.16; N, 0.31; Cl, 0.04, Mn, 0.10; 3-[Mn^III^]@RSNP, C, 26.86; N, 0.35; Cl; 0.05; Mn, 0.28; 5-[Mn^III^]@RSNP, C, 27.16; N, 0.37; Cl; 0.04, Mn, 0.48. Molar Mn/Si = 1.1%.

#### 2.2.2. Synthesis of Mesoporous [Mn^III^]@Carbon-Silica Nanoparticles ([Mn^III^]@CSNP)

[Mn^III^]@carbon-silica nanoparticles were synthesised by impregnation of the desired amount of [Mn^III^] compound (10, 30 and 50 mg for materials 1-[Mn^III^]@CSNP, 3-[Mn^III^]@CSNP and 5-[Mn^III^]@CSNP, respectively, which corresponded to around 1, 3 and 5 wt% of [Mn^III^] complex, respectively) with 1.00 g of carbon-silica mesoporous nanoparticles (CSNP) in acetonitrile (200 mL). The dispersion was stirred at room temperature for 24 h before filtration of the solid. The powder was then dried overnight at 80 °C. Anal. Found (wt%): 1-[Mn^III^]@CSNP, C, 30.33; N, 0.34; Cl, 0.02; Mn, 0.09; 3-[Mn^III^]@CSNP, C, 29.93; N, 0.42; Cl, 0.04, Mn, 0.13; 5-[Mn^III^]@CSNP, C, 31.06; N, 0.53; Cl, 0.05; Mn, 0.44. Molar Mn/Si = 0.7%.

#### 2.2.3. Synthesis of Mesoporous [Mn^III^]@Silica Nanoparticles ([Mn^III^]@SNP)

[Mn^III^]@silica nanoparticles were synthesised by impregnation of the desired amount of [Mn^III^] compound (10, 30 and 50 mg for materials 1-[Mn^III^]@SNP, 3-[Mn^III^]@SNP and 5-[Mn^III^]@SNP, respectively) with 1.00 g of SNP in acetonitrile (200 mL). The dispersion was stirred at room temperature for 24 h. The solid was then filtered and dried overnight at 80 °C. Anal. Found (wt%): 1-[Mn^III^]@SNP, C, 0.45; N, 0.26; Cl; 0.01, Mn, 0.09; 3-[Mn^III^]@SNP, C, 1.05; N, 0.33; Cl, 0.01; Mn, 0.26; 5-[Mn^III^]@SNP, C, 1.50; N, 0.34; Cl, 0.01; Mn, 0.40. Molar Mn/Si = 0.34%. 

### 2.3. Synthesis of [Mn^II^]@NP

The same procedure as for the incorporation of the Mn^III^ complex was followed using this time the Mn^II^ compound, also dissolved in acetonitrile. Anal. Found (wt%): 1-[Mn^II^]@RSNP, C, 27.21; N, 0.32; Mn, 0.05; 3-[Mn^II^]@RSNP, C, 26.66; N, 0.30; Mn, 0.17; 5-[Mn^II^]@RSNP, C, 27.08; N, 0.39; Mn, 0.24. 1-[Mn^II^]@CSNP, C, 29.46; N, 0.40; Mn, 0.03; 3-[Mn^II^]@CSNP, C, 30.58; N, 0.38; Mn, 0.24; 5-[Mn^II^]@CSNP, C, 30.93; N, 0.40; Mn, 0.38. 1-[Mn^II^]@SNP, C, 1.32; N, 0.19; Mn, 0.10; 3-[Mn^II^]@SNP, C, 1.76; N, 0.26; Mn, 0.24; 5-[Mn^II^]@SNP, C, 2.08; N, 0.29; Mn, 0.32. 

### 2.4. Characterization 

Chemical analyses (C, H and N) were performed by the CNRS Institute of Analytical Sciences in Lyon, France. *Carbon quantification*: the sample, sealed in silver capsule, was dropped into a flow of oxygen, the unit combustion was held at 1050 °C and followed by post combustion in a furnace at 850 °C containing copper oxide. The flash combustion allowed for the complete transformation of the carbon into carbon dioxide, which was quantified using a non-dispersive infrared detector. *Nitrogen quantification*: the sample, sealed in silver cup, was dropped in a flow of helium-oxygen in the combustion unit previously described, where nitrogen was converted into nitrogen oxides. The flow of gases was conveyed through a tube filled with copper wires, where nitrogen oxide was reduced into pure nitrogen, and quantified on a Thermo Conductivity Detector (TCD). *Total chlorine quantification* was performed using an Automatic Quick Furnace AQF100 (Mitsubishi Chemical Analytech, Yamato, Japan) connected to an Ion Chromatography ICS 1100 (Thermo Fisher Scientific, Sunnyvale, CA, USA). From 2 to 5 mg of sample previously weighed in a ceramic sample boat were introduced into a furnace maintained at 1000 °C in an oxygen-argon flow. Chlorine was then extracted from the gas stream and trapped (Cl_2_) in a hydrogen peroxide solution; 25 µL of this solution were directly injected into the ion chromatic integrion for analysis. *Metal analyses* were performed by Crealins in Lyon (France) using ICP-AES. *Infrared spectra* were recorded on KBr pellets in the 4000−400 cm^−1^ range with a Genesis Series FTIR^TM^ spectrometer (ATI Mattson, DeKalb, IL, USA). *Unpolarized Raman spectra* excited with 532 nm of a continuous wave solid-state laser were recorded with a LabRam HR800 spectrometer (Horiba^TM^, Kyoto, Japan) with a 100-fold magnification. Laser power was a threat for these samples because they strongly absorbed the light. Consequently, we paid a particular attention to avoid sample degradation by working with laser mean power under 100 µW. Typical collection time was 5 minutes for the first or the second order of each spectrum. For unpolarized UV Resonant Raman Spectroscopy (UVRRS) measurements the sample was excited with a 244 nm Laser and the data were collected with a dedicated Horiba^TM^ LabRam HR800 spectrometer with a 40-fold magnification. The laser source was a frequency doubled argon ion laser. Working with continuous mean power under 100 µW was not enough to preserve the sample. We had to spread the powder on the glass slide surface and rotate it with a motorized microscope turntable. By this way, the laser power was distributed on a larger surface and the Laser spot was always focused during the light collection (3 or 5 min). *Thermogravimetric analyses (TGA)* were performed with a STA 409 PC Luxx device (Netzsch, Selb, Germany) under aerobic conditions with a 10 °C/min temperature increase. *Nitrogen sorption isotherms* at 77 K were performed with a Belsorp Max volume device (Microtrac BEL Japan, Japan) on solids that were dried under vacuum overnight at 80 °C. The values after the incorporation of the manganese compounds were corrected considering the results of the elemental analyses to pass from results per gram of total material (including the complex) to results per gram of support (without the complex), which allowed us a direct comparison between samples loaded with different amount of manganese compounds. The *pH measurements* were carried out in a water solution using a PHM 210 Standard pH meter (Radiometer Analytical–Hach, Loveland, CO, USA) and a PHC 300 6 L-9 electrode. EPR spectra were recorded using an ESP-300E spectrometer (Bruker, Billerica, MA, USA) with a frequency of 9.4 GHz (X band) at the “Unitat de Mesures Magnètiques” (Universitat de Barcelona, Barce;ona, Spain). *Magnetic susceptibility* (χ_M_) measurements (2−300 K) were performed in a MPMS XL5 SQUID Magnometer (Quantum Design, Les Ulis, France) at the “Unitat de Mesures Magnètiques” (Universitat de Barcelona), using a field of 200 G. Simulations of the magnetic behaviour were performed using the PHI program [25]. *Scanning Electron Microscopy images (SEM)* were recorded using a Supra 55-VP microscope (Zeiss, Oberkochen, Germany). *Transmission Electron Microscopy (TEM)* images have been performed on a 2010F instrument (JEOL, Tokyo, Japan) at 200 kV. Samples for the TEM characterization were ground and directly placed on a copper grid coated with holey carbon film. *Light absorption* measurements were performed on a Cary 100scan UV-visible spectrophotometer from Varian-Agilent (Santa Clara, CA, USA). *Luminescence spectra* were measured by using a Horiba Jobin Yvon SPEX Nanolog fluorescence spectrofluorimeter equipped with a three-slit double-grating excitation and emission monochromator with dispersions of 2.1 nm/mm (1200 grooves/mm). Steady-state luminescence was excited by unpolarized light from a 450 W xenon CW lamp and detected with a red-sensitive R928 photomultiplier tube (Hamamatsu Photonics, Hamamatsu City, Japan) for solid-state measurements. Spectra were reference-corrected for both variation of the excitation source light intensity (lamp and grating) and the emission spectral response (detector and grating). The excitation wavelength used for all samples was 362 nm. 

## 3. Results and Discussion

### 3.1. Synthesis of the Materials Using Three Types of Mesoporous Nanoparticles

We chose two manganese complexes possessing 2,2′-bipyridne (bpy) and 9-antracenecarboxylate (AntCO_2_) as ligands in order to incorporate them into different types of nanoparticles. One of the complexes is a tetranuclear Mn^III^ cluster [Mn^III^_4_(μ-O)_2_(μ-AntCO_2_)_6_(bpy)_2_(ClO_4_)_2_] with a butterfly core [24,26]. The second is a dinuclear Mn(II) complex, where the two Mn centers are bridged by two carboxylate groups and one water molecule [{Mn^II^(bpy)(AntCO_2_)}_2_ (μ-AntCO_2_)_2_(μ-OH_2_)]. For the latter, the dinuclear species is the main compound in solution. However, depending on the synthesis conditions, a 1D molecular chain can also form and be isolated as a solid [24].

**Scheme 1:**
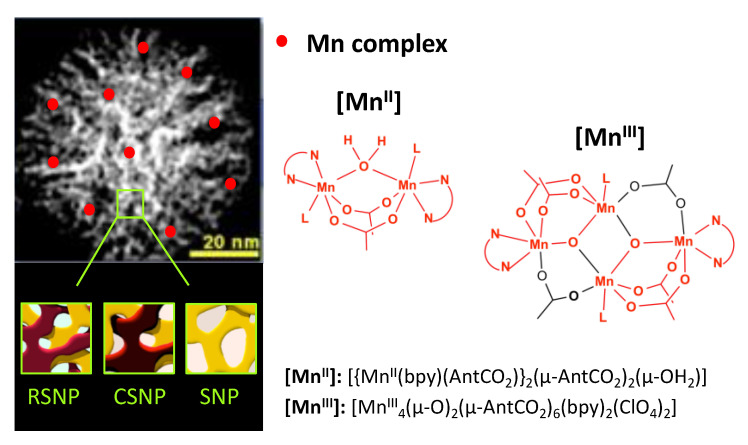
Synthesis of the three types of mesoporous nanoparticles containing the manganese complexes. RSNP: resol-silica nanoparticles, CSNP: carbon-silica nanoparticles, SNP: silica nanoparticles. L: ClO_4_^−^ or AntCO_2_^−^ ions and N-N: bipyridine ligand.

In order to compare the effect of the matrix composition on the final material, the manganese complexes were incorporated into three different types of mesoporous nanoparticles: resol-silica, carbon-silica and pure silica nanoparticles (RSNP, CSNP and SNP, respectively, Scheme 1). These three types of nanoparticles were synthesized using a triphasic syntaxic approach developed by Zhang and co-workers, which yields nanoparticles with a similar porosity network but with a different composition [23]. These characteristics were chosen in order to facilitate the comparison between the final materials. The synthesis of the nanoparticles requires: (i) tetraethoxysilane (TEOS) as silica source, (ii) resorcinol (1,3-dihydroxybenzene) and formaldehyde (methanal) as the two precursors of the resol (also called resorcinol-formaldehyde resin) network, (iii) cetyltrimethylammonium bromide (CTAB) as micellar templating agent, (iv) triethanolamine (TEA) to adjust the pH and to favour dendritic porosity, and (v) ethanol as a co-solvent for kinetic and thermodynamic purposes [23]. Indeed, ethanol slows down the polymerisation speed of the resin, which is crucial for the three-body syntax to happen, and alters the superficial tension of the micelles in order to control the particle diameter [27]. After this, the hybrid resol-silica nanoparticles obtained can be thermally treated to obtain either pure mesoporous silica nanoparticles or hybrid carbon-silica nanoparticles. Silica nanoparticles required further calcination of the RSNP in air at 550 °C to remove the organic phase. By contrast, CSNP were obtained by carbonization of the RSNP under N_2_ atmosphere at 900 °C in order to decompose the organic part in carbon without any degradation into CO_2_.

The syntaxic approach relies on complex interactions between three phases, namely, the inorganic matrix composed of silica anionic tetrahedra (I^−^), the positively charged micelles of surfactant (S^+^, here cetyltrimethylammonium cations CTA^+^), and the organic polyphenolates from the resol polymer (O^−^). In order to obtain a homogeneous dendritic system for the two hard phases, mastering the kinetics of the reaction is a priority. The two main factors that influence the condensation rate of the materials are their concentration and the pH of the suspension. In classic basic conditions (pH~10), the condensation rate of the polyphenol is lower than the one of the silica, leading to non-porous resol spheres coated with a silica-shell [23]. At neutral pH, the opposite happens and the two phases condense in separate aggregates. However, when the conditions are carefully tuned (pH ~ 8), both of the condensation reactions happen at the same rate, leading to a cooperative growth system: syntaxy. In these conditions, both silica tetrahedrons and polyphenolic anions are adsorbed on the surface of the micelles and develop their network at the same time. During these nucleation and growth steps, the two phases have to interact constantly with each other, thus leading to two porous phases, one being the opposite of the other [23]. 

The incorporation of the manganese complexes inside the three types of nanoparticles was done by impregnation of 1, 3 and 5 wt% of the corresponding Mn^III^ or Mn^II^ complex (see experimental section). The amount of Mn was determined by ICP and ranged between 0.03 to 0.5 wt%.

### 3.2. Characterization of the Nanoparticles 

#### 3.2.1. Infrared Spectroscopy

In all particle types, the bands characteristic of silica were clearly visible (Figure S1). Firstly, two bands that can be attributed to stretching modes: a wide intense band between 1240 and 1040 cm^−1^ corresponding to Si-O-Si vibrations, and a moderate band at 970 cm^−1^ for silanol functions. Secondly, two less intense bands at 800 cm^−1^ and 465 cm^−1^ were observed, which are respectively the bending and rocking modes of Si-O-Si bonds. Between 1620 and 1640 cm^−1^ the bending mode of molecular H_2_O shows that water quickly adsorbed in the particle’s cavity, even in the dried material. In the hybrid resol-silica nanoparticles, a series of more structured bands appeared in the 1345–1550 cm^−1^ range. They match aromatic sp^2^ C=C vibrations and the C–O stretching mode of the polyphenolic network. Moreover, in the 2930–2855 cm^−1^ area, two small bands representative of the C_sp2_–H and C_sp3_–H stretching modes were observed. They are characteristic of the CTA^+^ surfactant. This shows that even after several washings in acid solution, a small part of the templating agent remained inside the material.

Finally, in the carbon-silica nanoparticles, the bands of the resol polymer were replaced by a wider and less intense band between 1500 and 1670 cm^−1^ that is attributed to the stretching mode of sp2 C=C bonds. The water contribution to the spectrum (bands at 3500 and 1800 cm^−1^) was much smaller. The total absence of Si-C vibration peaks indicates that the silica and carbon phase share no chemical bonds. 

Due to the relatively low load of the complex inside the nanoparticles, the infrared spectrum of [Mn^III^]@NP and [Mn^II^]@NP did not show much difference from the raw material (See some examples in Figure S2). However, the weak bands found at around 738 cm^−1^ and 765 cm^−1^ denote the presence of the compound inside the particles. The 738 cm^−1^ band is assigned to ν(MnO), while the 765 cm^−1^ band is attributed to aromatic δ(CH) vibrations of the anthracenecarboxylate ligand. Nevertheless, it was not possible to unequivocally attribute this vibration to the bridging anthracenecarboxylate function or the the μ-oxo bridge. 

#### 3.2.2. Raman Spectroscopy

**Figure 1:**
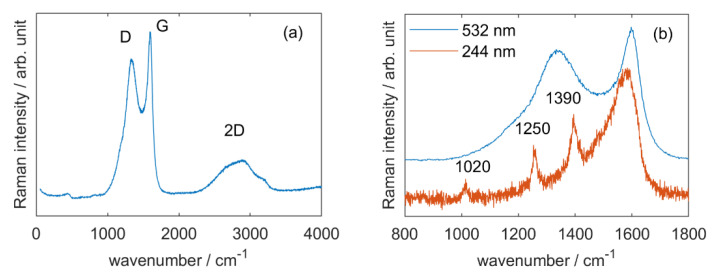
Raman spectra of (**a**) CSNP (λ_ex_ = 532 nm) and (**b**) Visible (blue line) and UV (red line) Raman spectra of [Mn^III^]@CSNP (λ_ex_ = 532 and 244 nm). The Raman spectra were corrected with a linear baseline to fit D and G bands with a Lorentzian function and a Breit-Wigner-Fano function, respectively [34]. Then, the intensity ratio I_D_/I_G_ was determined at 532 nm. A very low laser power (~80 µW) was used at 532 nm to preserve the sample from any degradation. Deep UV Raman spectroscopy was performed according the protocol described by Montagnac et al. [35].

The study of CSNP using Raman spectroscopy was first performed with an excitation wavelength of 532 nm (Figure 1a). Three bands characteristic of graphitised materials were observed. The G band at 1600 cm^−1^ corresponds to the in-plane stretching vibrations of graphene, implying the C_sp2_=C_sp2_ bonds. The D band at 1330 cm^−1^ is attributed to structural defects in the graphene layers that disrupt the normal stretching mode. In our case, this band can be attributed to the presence of graphite-oxide, which is the result of the partial decomposition of the resol-based material. Finally, the G_0_ (or 2D) band at 2800 cm^−1^ results from a two-phonon resonance phenomenon that occurs between the graphene sheets. The graphitisation rate of our material can be evaluated by comparing the intensity ratio between the D and G bands to the one of pure graphite material. We found a ratio I_D_/I_G_ = 0.87 compared to 0.16 for pure graphite [28,29]. This value is well in the range of other graphite oxide materials [28].

Then the spectra of [Mn^III^]@CSNP was recorded using both visible and UV Resonant Raman Spectroscopy (UVRRS, Figure 1b). The D mode is dispersive; it varies with photon excitation energy, even when the G peak is not dispersive [30]. We used a deep UV laser (244 nm) to minimize the D band contribution and enhanced resonance Raman modes, currently the ones coming from the ligands of the Mn compound incorporated inside the nanoparticles. Three new bands were detected (1020, 1250 and 1390 cm^−1^) and the G band appeared larger (1580–1600 cm^−1^) than with the excitation in the visible range. The weak band at 1020 cm^−1^ can be attributed to deformations of aromatic rings (δ(ring)) [31,32], which gives us little information, considering that both the ligands and the support present aromatic rings. The 1250 cm^−1^ is however more interesting and corresponds to a combination mode of aromatic deformation (δ(ring)) and CH deformation(δ(CH)) [31,32]. As the support is normally fully saturated, it suggests the presence of the ligands. However, as defects are present in the graphite, this does not constitute sufficient proof. The 1390 cm^−1^ can be attributed to symmetrical stretching of the carboxylate groups (νs(CO_2_^−^)) [33] and indicates the presence of the anthracenecarboxylate. Finally, the widening of the 1600 cm^−1^ can be due to multiple vibration modes like C=N or C_2_O deformations [31], which can be attributed to the bipyridine or the presence of graphite oxide. All in all, the information provided by UVRRS show the presence of organic aromatic rings and carboxylate functions, strongly suggesting the presence of the ligands inside the nanoparticles.

#### 3.2.3. NMR Spectroscopy

^29^Si-NMR allows us to distinguish silicon atoms involved in siloxane bridges (Q_4_), single silanol (Q_3_) and geminal silanol groups (Q_2_) on the surface of silica. The resol-silica hybrid nanoparticles (RSNP) present a large amount of Q_3_ and Q_4_ species (respectively at −100 and −110 ppm), along with a small quantity of Q_2_ species (−90 ppm, Figure S3a). A fit using multiple Gaussian curves of the HPDEC NMR spectrum of RSNP gave a Q_2_/Q_3_/Q_4_ ratio of 10.7/60.9/28.4. This ratio shows that the silica phase was not fully condensed after the initial synthesis. When the particles were submitted to thermal treatments to yield either SNP or CSNP, the Q_3_ contribution to the signal was greatly diminished and only the Q_4_ peak was clearly visible. This was due to the condensation of surface silanol groups into SiO2, which occurs at high temperature.

^13^C-NMR spectroscopy was also performed, both on RSNP and CSNP (Figure S3b). The peaks corresponding to phenolic and alkyl carbons of the resol phase were identified for the RSNP. The CSNP spectrum changed compared to that of RSNP, suggesting that the thermal treatment under a N_2_ atmosphere modified the organic phase. 

#### 3.2.4. Thermogravimetrical Analysis (TGA)

An important mass loss (50–60 wt%) occurred between 250 and 650 °C for hybrid resol-silica, and between 500 and 750 °C for the corresponding carbon-silica material (Figure S4, TGA under air). This mass loss was attributed to the degradation of organic species. Interestingly, the slope of the mass loss corresponding to the degradation of carbon was steeper and occurred at higher temperatures for carbon silica nanoparticles. The increased stability of the latter system was relevant, considering the structure change from organic to inorganic carbon, a change that implies a different degradation mechanism.

An accurate estimation of the mass loss due to the manganese complex for [Mn^III^]@RSNP using TGA was not possible. This difficulty came from the low amount of the complex inside the material. When measured separately, the Mn^III^ compound decomposed between 180 °C and 540 °C and the Mn^II^ complex between 280 °C and 550 °C, leaving residual MnO_2_ in both cases. On the other hand, the presence of the manganese compound inside the nanoparticles catalysed the decomposition of the organic part of the material (Figure S5). Using the derivative mode, we observed that the last inflexion of the curves occurred at 506 °C for the empty RSNP, at 489 °C for the ones loaded with 10 mg of [Mn^III^], at 455 °C for 30 mg and at 436 °C for 50 mg. This was attributed to the amount of manganese oxides generated by the calcination that likely catalysed the degradation of the material. Indeed, the more complexes inside the particles, the more oxides were generated, and the faster the material was degraded. The same behaviour was observed for [Mn^II^]@RSNP materials (Figures S6 and S7). 

#### 3.2.5. Evaluation of the Size and Porosity

**Figure 2:**
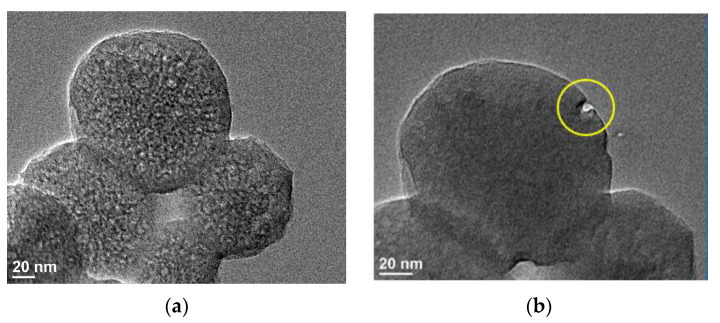
TEM images: (**a**) 5-[Mn^III^]@RSNP; (**b**) 5-[Mn^III^]@RSNP after EDX analysis, the cropped part is due to the electron beam action.

All the samples are well-defined spherical nanoparticles having a diameter of approximately 150 nm, with a low dispersion in terms of size and shape of the particle (Figure S8). The pores size is smaller than 10 nm, as observed in the TEM images for 5-[Mn^III^]@RSNP (Figure 2a). It appears also that the porous particles seem to be coated with a thin layer of polymer. This was confirmed by the degradation of the material when submitted to the focused electron beamed used for EDX. The resol network was damaged while the silica part was more resistant (Figure 2b). The fact that the beam can pierce through the layer confirms that it was mainly formed of polymers. The generation of this thin layer was not surprising as the same synthesis performed at higher pH yields core-shell resol@silica nanoparticles [23]. EDX analysis was performed at different parts of the nanoparticles. The average molar ratio Mn/Si measured by EDX was 1.0% for 5-[Mn^III^]@RSNP, which is close to the value calculated from elemental analysis and TGA of the bulk (Mn/Si = 1.1%). The amount of Mn was more important at the particle’s periphery (Mn/Si = 1.8%) than inside (Mn/Si = 0.6%), suggesting that the metal complex was staying close to the particle surface and only a part of them penetrated deep inside.

**Figure 3:**
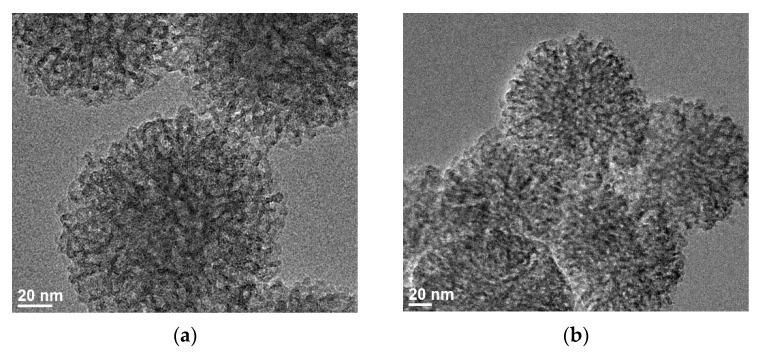
TEM images of (**a**) 5-[Mn^III^]@SNP and (**b**) 5-[Mn^III^]@CSNP.

The TEM images of 5-[Mn^III^]@SNP are presented in Figure 3a and Figure S9. The dendritic structure of the particles is clearly visible as well as the pores when zooming in. A statistical analysis on the TEM images showed that pores distribution was wide, ranging from 4.4 to 11.0 nm. These values were in agreement with the nitrogen sorption isotherm for this material *(vide infra*). Furthermore, the average Mn/Si molar ratio measured by EDX was 0.36%, in agreement with the value found by elemental analysis (Mn/Si = 0.34%).

TEM images of 5-[Mn^III^]@CSNP (Figure 3b and Figure S10) show that CSNP have are very similar in size and shape to RSNP. The amount of Si and O measured by EDX at different parts of the particle was close to the average value measured with elemental analysis. However, the Mn/Si molar ratio detected (0.3%) was lower than that measured by elemental analyses (0.7%), which suggests an inhomogeneous distribution of the complex inside the particle. Nevertheless, the Mn/Si molar ratio recorded at the particles edge (0.4%) was more important than in the center (0.2%), which suggests once again that the penetration of the manganese complex was not total and that the complex prefers to bind on the particle’s surface or the edge of the channels.

The study of the nitrogen sorption isotherms shows that the overall porosity of SNP is higher than the one of RSNP and CSNP, which is consistent with the thermal treatment that burned the organic phase away, thus leaving wider cavities (Figure S11 and Table S1). All types of nanoparticles present a sorption isotherm close to a type II at the adsorption branch, and definitively to a type IV at the desorption branch, according to the IUPAC classification [36]. In fact, the situation is better analyzed on the silica nanoparticles having higher por volumes than the hybrid ones (RSNP and CSNP). At a second glance, the adsorption branch should be indeed considered of type IV, taking into account the slow increase of the adsorbed volume above p/p^0^ = 0.42, assigned to a progressive capillary condensation and to a variety of mesopore sizes. In all the nanoparticles the hysteresis revealed by the desorption branch concerns a small fraction of the pore volume (*ca.* 10%) that could be due to some bottle-neck effect. The smaller pore volumes of the hybrid RSNP and CSNP are consistent with the location of the resol or carbon phase inside the pore of the dendritic silica phase. All these characteristics are typical of the entangled dendritic phases in these hybrid nanoparticles [23].

While the pure silica and hybrid resol-silica materials have a C_BET_ value in the usual range for silica materials (C_BET_~100), this value is much higher for the hybrid carbon-silica material (C_BET_ = 900). Indeed, the C value reflects the interaction between polar silanol groups and apolar N_2_ species. This interaction induces a dipolar moment between the molecules. A similar effect is likely to appear with the phenol group of the resol phase, and this seems to be confirmed by the C_BET_ value measured for resol-silica nanoparticles (C_BET_ = 102). By contrast, and as reported by other authors, the graphitised surface of the carbon-silica nanoparticles appears to stabilise free carbon radicals [37,38,39]. According to the high C_BET_ value observed (900), these radicals seem to contribute to a stronger interaction with N_2_ molecules, highly increasing the N_2_ molecules affinity for the material.

Upon incorporation of the Mn complexes into RSNP the sorption isotherm was not significatively modified, with a mesoporous porous volume of around 0.3 cm^3^/g (Figures S12 and S13 and Tables S2 and S3). This might come from the flexible nature of the polyphenol with the ability to adapt, to a certain point, to N_2_ pressure. For 3-[Mn^III^]@RSNP and 1-[Mn^III^]@RSNP the volume was slightly higher than for the empty nanoparticles. Considering the mechanical properties of resorcinol-formaldehyde resin, which is very flexible and has a tendency to inflate with the solvent, the insertion of the manganese complex may contribute to change the NP pore morphology. Either by pushing the flexible resol walls or by dragging the polymer in the periphery or outside the silica network. An important inter-grain porous volume was observed in all the cases, the total porous volume being higher than 1 cm^3^/g. The size of the pores, measured with BJH, was comprised between 1.4 and 4 nm and in some cases up to 7 nm, as expected for a dendritic porosity [23].

N_2_-sorption isotherms for Mn@SNP showed a clear decrease of the mesoporous volume after incorporation of the manganese compound from 0.65 cm^3^/g to 0.44 cm^3^/g in the samples with the higher content of Mn complex (Figures S14 and S15 and Tables S4 and S5). Again, a high inter-grain porous volume was observed in all the cases and the total porous volume (V_t_) was higher than 1 cm^3^/g. The pore average diameter and dispersion were almost identical among loaded and empty SNP. It was not very surprising: the empty SNP had a high pore volume, which was only partially filled upon the incorporation of the manganese compounds. These figures also strengthened the idea that in hybrid resol-silica particles, modification of the characteristics of the cavities comes from the polymer phase and not from the more rigid silica phase. For Mn^III^ compounds, a drop in pore volume and internal specific surface was clearly shown when the first amount of compound was added (10 mg). Then, with larger amounts (30 and 50 mg), the loss in pore volume and disponible area was much less important. This result supports the idea that the initial loading takes place on the surface and entrance of the pores network, partially sealing the channels and that further complexes added may have trouble penetrating the particles and probably partially break to do so. On the contrary, for Mn^II^ compounds, a decreasing trend in the specific surface area was clearly showed. The loss of 7 to 33% of the internal area compared to SNP and the diminution of the external area of up to 5% show that the compounds are located inside the particle’s cavities. This area loss was higher when the amount of compound inserted increased, compared to [Mn^III^]@SNP. It shows a better insertion of [Mn^II^] molecules, which progressively fill the pores, whereas [Mn^III^] seems to impede them quicker. On the other hand, the characteristic diameters measured did not change significantly, indicating that the pore structure stayed the same.

N_2_ sorption isotherm for carbon-silica nanoparticles filled with manganese compounds showed the greatest pore volume decrease (Figures S16 and S17 and Tables S6 and S7). The C value also drastically evolved with the insertion of the complexes. For example, for [Mn^III^]@CSNP materials, C started at 900 and quickly dropped to 468 to finish at 291 for the most loaded sample. This shows either that the presence of [Mn^III^] has a strong impact on N_2_ affinity with the materials or that its insertion changes the inner configuration of the particle. The decrease of Vmeso to 11% for 5-[Mn^III^]@CSNP and the slight increase of the maximum diameter from 4.8 nm for empty particles to 5.4 nm for 3-[Mn^III^]@CSNP and 5-[Mn^III^]@CSNP suggests a change in the porosity. A similar behaviour was observed for [Mn^II^]@CSNP, the total pore volume diminishing even more than for [Mn^III^] incorporation upon increasing the compound loading: 10% for 1-[Mn^II^]@CSNP, 40% for 3-[Mn^II^]@CSNP, and 76% for 5-[Mn^II^]@CSNP. On the other hand, the pore’s average and extremal diameter did no change significantly, indicating a stability of the walls and that a significant amount of space was still available inside the nanoparticles.

#### 3.2.6. Study of the Integrity of the Complex inside the Nanoparticles and Estimation of the Load of Complex

In order to check the integrity of the complex the study of the magnetic properties of the materials was performed. The magnetic behaviour of the support itself was first studied. SNP and CSNP presented a significant answer to magnetic field, contrary to RSNP (Figure S18a). Indeed, it was possible to assign these signals to the presence of paramagnetic defects inside the nanoparticle matrix, induced by the thermal treatment. These impurities probably originate either from carbon or from carbon induced defects in the silica matrix. Indeed, for other [Mn]@silica materials reported in the literature [40,41,42,43], the silica support does not present such a magnetic answer. Actually, they were either prepared without thermal treatment [40,41,42] or in the presence of smaller number of organic molecules during the calcination process [43]. The presence of such impurities was supported by the detection of carbon radicals in the CSNP material using electron paramagnetic resonance (EPR) spectroscopy (Figure S18b). The presence of these radicals has been documented for graphitised mesoporous systems [39] and nanoparticles [37,38].

The study of the magnetic properties of [Mn]@NP materials was not straightforward. Unfortunately, the simple subtraction of the blank for SNP and CSNP led to a strong distortion of the signal. Moreover, their curves display shoulders characteristic of the presence of dioxygen impurities inside the porous matrix. It should also be noted that the amount of manganese compound was quite small compared to the mass of the sample (between 2.9% and 4.6%), weakening the magnetic answer and making the signal more sensitive to the effect of the impurities. As a consequence, not all the data could be analysed. The signal of [Mn^II^]@RSNP and [Mn^III^]@RSNP was however clean enough to make observations and interpretations (Figure S19). As the magnetic answer of RSNP alone was negligible, the signal detected for [Mn]@RSNP could be attributed to the Mn entities loaded inside. Some conclusions could be drawn. First, χ_M_T *versus* T curves of both [Mn^II^]@RSNP and [Mn^III^]@RSNP decreased as temperature diminished. This decrease was at low temperatures, indicating a weak antiferromagnetic interaction between the manganese ions. However, χ_M_T did not reach zero for the lowest temperature measured. In order to explain the shape of these χT graphs, several simulations were carried out using the PHI program (spin Hamiltonian for a dinuclear system H = −2JS_1_·S_2_) [25]. It was not possible to reproduce the graph considering either only the zero-field splitting (ZFS) of the Mn ions or only the magnetic interaction. Therefore, both parameters were necessary to obtain a good simulation. From the different simulations we can propose that: (1) the magnetic data of the [Mn^III^] system inside the RSNP suggests that the tetranuclear entity was broken and a dinuclear entity was likely formed (Scheme 1) since a weak antiferromagnetic coupling typical of Mn^III^ dinuclear species was observed [44] instead the expected magnetic behaviour of the Mn^III^_4_ butterfly complex [24]; (2) in both cases (Mn^II^ and Mn^III^) a dinuclear entity was considered as the main species and a weak magnetic interaction was found between the Mn ions for both [Mn^II^]@RSNP and [Mn^III^]@RSNP samples; (3) for the [Mn^III^]@RSNP system a dinuclear unit was considered and typical values of ZFS of the Mn^III^ ions were used, obtaining a weak antiferromagnetic magnetic exchange between the Mn sites (2J = −1.2 cm^−1^) and a D_Mn_ quite important (D_Mn_ = −3 cm^−1^), as usual for Mn^III^ complexes [44,45]; (4) the simulation of [Mn^II^]@RSNP did not reproduce well the behaviour of experimental data at low temperatures, suggesting a weak magnetic coupling constant (2J = −1.2 cm^−1^) [46] and an orthogonal distortion higher than expected for Mn^II^ ions (D_Mn_ = 1 cm^−1^). As a conclusion, from these results we can expect that a dinuclear entity was present inside the nanoparticles in all the cases. From these results together with the chemical analyses and TGA the load of the manganese complex was estimated (Table 1). It was lower than the theoretical value in almost all the samples. 

**Figure 4:**
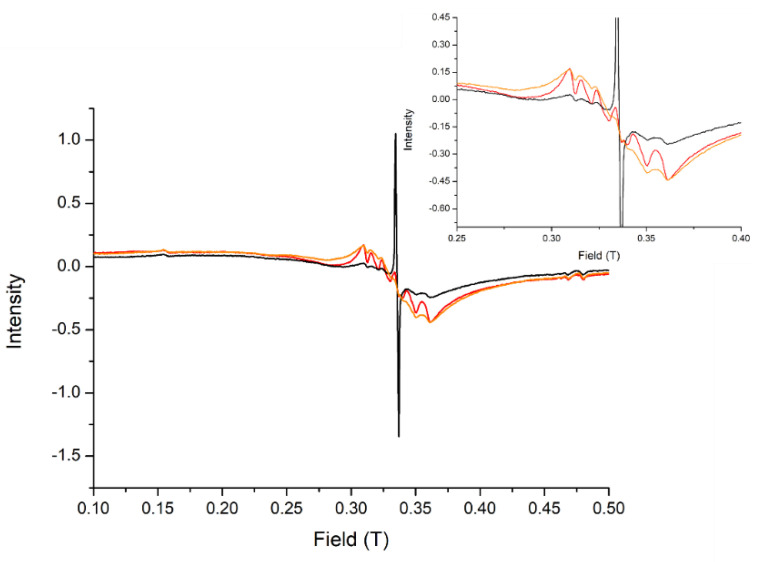
X-band EPR spectrum of [Mn^II^]@RSNP (red line), [Mn^II^]@SNP (orange line) and [Mn^II^]@CSNP (black line) measured at 77 K. Inset: zoom of the main signal.

The EPR spectra of the nanoparticles loaded with Mn^II^ complexes were recorded at low temperature (77 K). They show a six-line signal typical of Mn^II^ (Figure 4). This kind of spectrum is usually found in solution, which indicates that inside de nanoparticles the complexes are distributed in the pores and the interactions between them are scarce [40,41,43]. The signal can be assigned to dinuclear Mn^II^ species with weak magnetic interactions between the metallic centers and hyperfine coupling due to the manganese nuclear spin (I_Mn_ = 5/2). The spectrum with the best resolution was observed for the RSNP, while spectrum for SNP showed a lower resolution, probably due to the presence of impurities in the support (*vide supra*). The spectrum of the CSNP showed a sharp band characteristic of a radical (*vide supra*) together with the six smaller bands dues to the [Mn^II^] species. These kinds of spectra are typical of dinuclear systems with weak coupled Mn^II^ ions [46]. However, it was not possible to conclude if some structural changes like ligand exchange or Mn-Mn distance variation occurred in the Mn^II^ complex upon internalisation.

### 3.3. Study of the Luminescent Properties of the Materials

**Figure 5:**
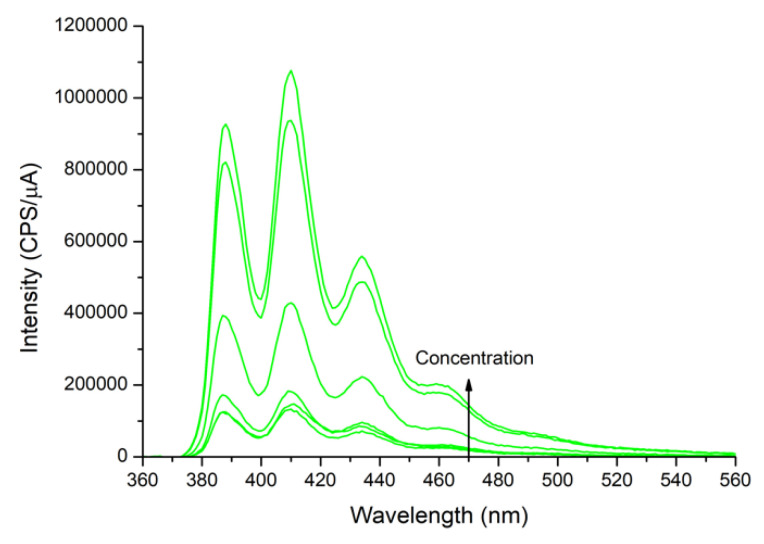
Emission spectra of [Mn^II^] compound in ethanol with increasing concentrations (~10^−7^ to 10^−6^ mol/L). Excitation wavelength: 362 nm.

**Figure 6:**
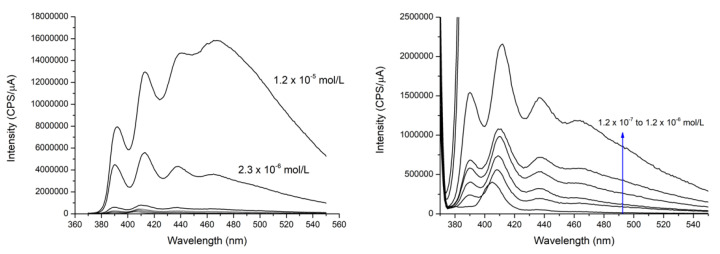
Emission spectra of [Mn^III^] in ethanol with concentrations from 1.2 × 10^−5^ to 1.2 × 10^−8^ mol/L. Excitation wavelength: 362 nm. All spectra were recorded with two different spectrometers settings one adapted to the highest concentrations, the other to the lowest ones. The spectra displayed are the ones with the best resolution to saturation ratio, hence the change of unit scale.

In order to determine the optical properties of the hybrid materials, we first studied the optical properties of the molecular compounds (Figure 5 and Figure 6). The absorption and emission spectra of 9-anthracene carboxylic acid are well described in the literature (see for example Abdel-Mottaleb et al.) [17]. The band centred at 254 nm in the absorption spectrum corresponds to the ^1^A → ^1^Bb transition (in Platt notation), and the bands at 384 nm, 365, 347 and 330 nm are attributed to the A^1^ → L^1^ transition. As expected, the emission spectrum mirrors the latter bands with the separation of the Stokes shift. These bands, found at 390, 412, 438 and 464 nm, correspond to the L^1^ → A^1^ transition [17,20]. The appearance of a large and intense band at 470 nm can occur in case of a change in concentration of the solution, a modification of the solvent polarity, or the pH value. A discussion on the effect of these parameters was presented in a recent work by Rowe and collaborators [20]. By studying the effect of the positions of the carboxylic acid groups on anthracene carboxylic diacids, they came to the conclusion that the structure of the emission is strongly affected by the contribution of the carboxylic acid groups to the excited state of the molecule. Effects that prevent the contribution of the carboxylic acid groups (hindrance of the rotation angle, deprotonation or inductive effects) favour the contribution of the anthracene ring atoms to the excited states, therefore structuring the emission spectrum. On the other hand, if the contribution of the carboxylic acid to the excited state is promoted (by factors opposite to the ones previously cited), the bands widen and lose their structure. A so-called exciton band is therefore observed. Moreover, effects that stabilise the energy of the main excited state (like the solvent polarity or the functionalisation of the ring) tend to favour a bathochromic shift (in the range of 20–30 nm for the examples studied by Rowe) [20]. 

The manganese complexes here studied exhibited a strong luminescence in ethanol solution. The [Mn^II^] compound is a dinuclear complex that can crystallize either as a dinuclear entity or as a chain, whereas the [Mn^II^] compound is likely a tetranuclear entity *(vide supra).* Regardless of the concentration, the [Mn^II^] emission spectrum always had the same shape, with distinctive anthracene-like vibronic bands at 390, 412, 438 and 462 nm (Figure 5). On the other hand, [Mn^III^] emission was strongly concentration-dependent (Figure 6). Its spectrum also displayed vibronic bands at 390, 412 and 438. However, the spectrum presented a large band with a maximum located between 460 and 468 nm, whose intensity increased with concentration (Figure 6). It is interesting to notice that the maximum of this band was always below the 470 nm observed in ethanol for the free molecule, which tends to dismiss the hypothesis of the ligands decoordination in solution [17].

After the insertion of the manganese complexes inside the nanoparticles, fluorescence was still detected but several questions arose. Is the emission of the complexes modified by their environment inside the nanoparticles? Are potential modifications of the complexes structure detected through fluorescence spectroscopy? Do the complexes leach out in the solution during luminescence measurements? This section attempts to answer these questions.

**Figure 7:**
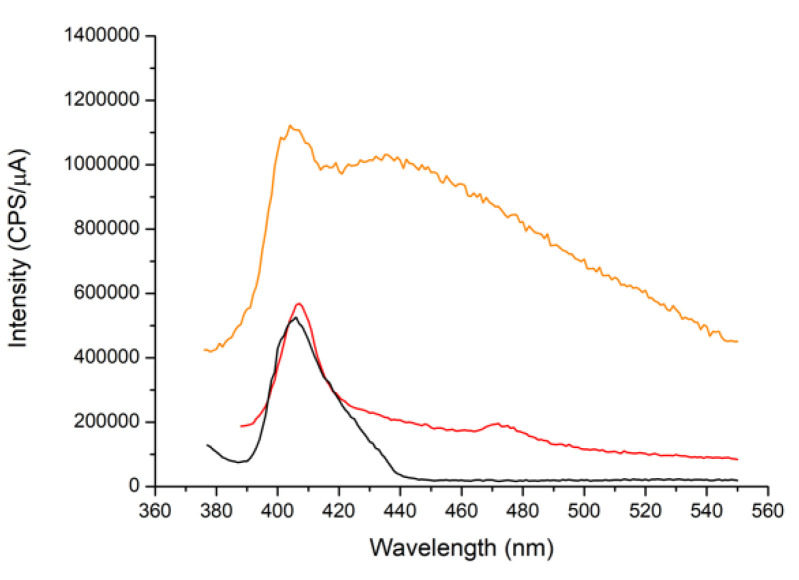
Emission spectra of RSNP (red line), SNP (orange line) and CSNP (black line) suspension in ethanol (250 mg/L) with a 362 nm excitation wavelength.

First, studying the emission spectra of the unloaded nanoparticles in ethanol suspension (250 mg/L) was needed in order to see how they contribute to the luminescence of the whole hybrid material (Figure 7). The particles were excited at 362 nm, which was our working wavelength for both manganese complexes. They all displayed the same 407 nm band that comes from the silica phase. This large band can be found in different siliceous materials, usually quartz crystals and silica aerogels. It is supposed to come from defects inside the silica structure and many defect-related mechanisms have been proposed (non-bridged oxygen hole centers, carbon impurities, nitrogen centred effects, charge transfer mechanisms or surface Si-OH states) [47,48]. For silica nanoparticles, usually prepared in basic conditions, this is rarer. Jakob and Schmedake reported this type of luminescence for nanoparticles modified with (3-aminopropyl)triethoxysilane (APTES) [47]. They linked this phenomenon with the amount of carbon introduced with APTES inside their particles, and hence to the carbon impurities generated during calcination. In a later article they showed that an emission transfer with lanthanides ions was possible and reduced the fluorescence of their material [49]. However, they did not observe any luminescence before thermal treatment of their particles, contrary to our case. In the emission spectrum of SNP, the band maximum was followed by a large shoulder with a maximum around 435 nm (Figure 7). For the other types of nanoparticles, this shoulder was much narrower, and did not have a secondary maximum. It was more akin to an asymmetric deformation of the main 407 nm band.

Finally, for RSNP, another band was recorded around 475 nm and attributed to the luminescence of the resol polymer [50,51]. This band disappeared for CSNP, which is a good indicator that the polymer was degraded to amorphous carbon. Multiple interpretations can be given for the reduced intensity of RSNP and CSNP compared to SNP. The silica phase may transfer some energy to the organic phase, thus quenching the emission process. It is also possible to consider that if other materials require a thermal treatment for luminescence to be observed, the effect of the defects present in our materials would be reinforced with the calcination process. This, however, would not explain why CSNP emit as much as RSNP. The intensity change could also be due to a different interaction of the materials with the solvent molecules. Hybrid mesoporous nanoparticles are more hydrophilic than the pure silica ones, and are thus expected to retain more solvent. However, given the multiple possible origins of this phenomenon of luminescence and the limited data we collected about it, it is impossible to give a definitive answer.

Interestingly, the emission of all particle types was largely quenched when the manganese complexes were inside. And even the emission recorded for [Mn]@SNP and [Mn]@CSNP looks much more like the spectrum of the complex than the spectrum of the nano-vector material (Figure S20), meaning that its contribution was greatly reduced overall. Moreover, these graphs demonstrated that at least a minimal number of complexes remained either inside or on the particle surface, enough to extinguish the nanoparticles emission. As a consequence of this discussion, in the following paragraph all the [Mn]@NP emission spectra did not receive any blank subtraction, as we considered that the luminescence of the nanoparticles themselves was completely quenched. 

**Figure 8:**
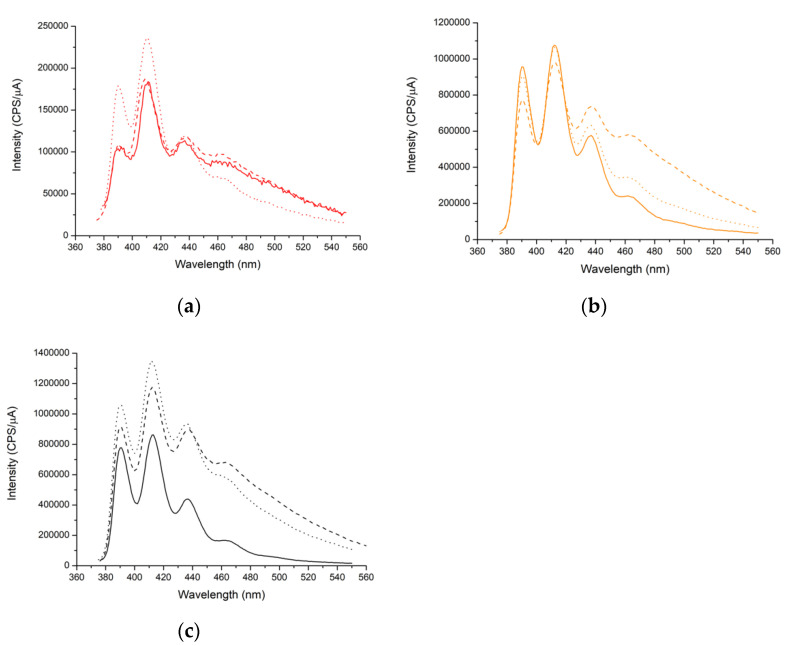
Emission spectra of [Mn^II^]@NP for each NP type (full line) compared to the emission spectra of the supernatant after centrifugation (dashed line) and the filtrate (dotted line): (**a**) 5-[Mn^II^]@RSNP, (**b**) 5-[Mn^II^]@SNP and (**c**) 5-[Mn^II^]@CSNP.

**Figure 9:**
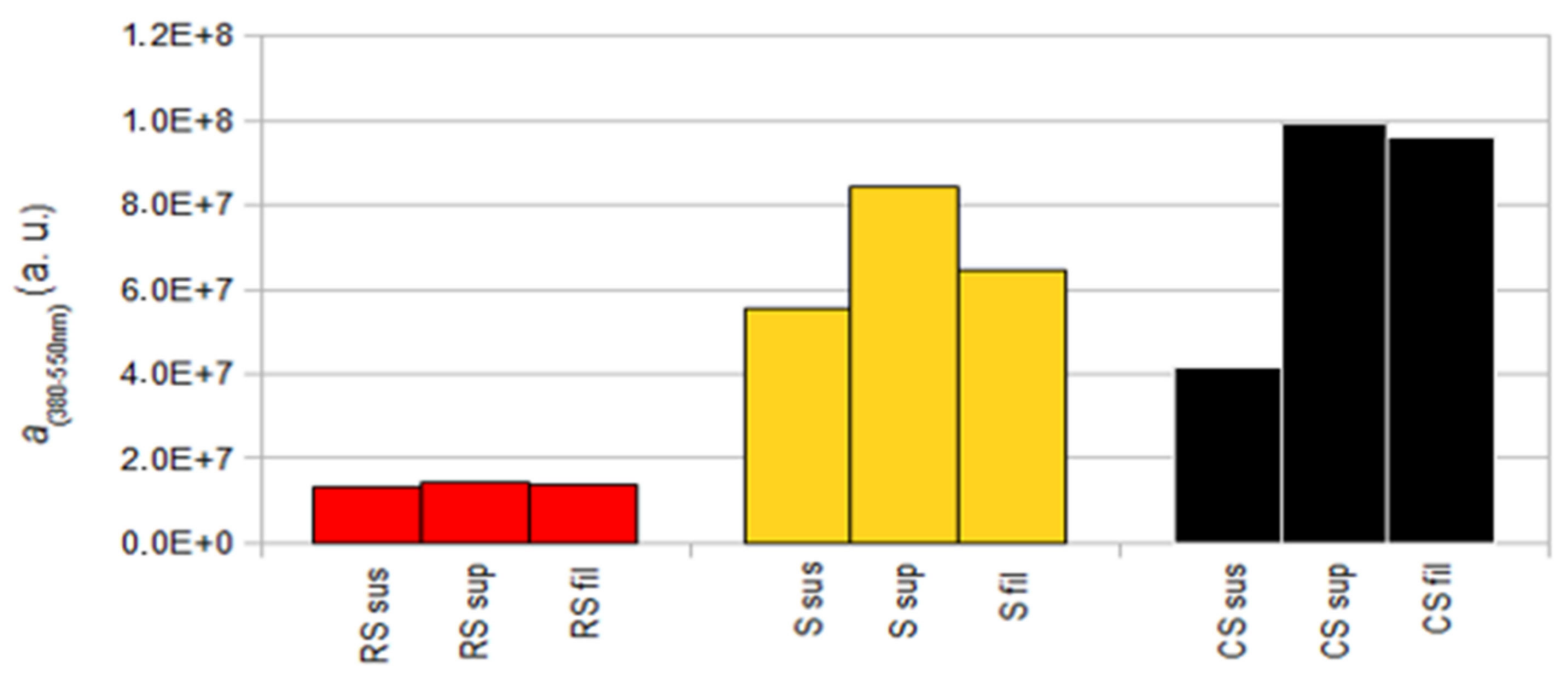
Value of the area below the emission curve between 380–550 nm for 5-[Mn^II^]^2.9^@RSNP (5.5 × 10^−7^ mol/L) (RS—red), 5-[Mn^II^]^3.9^@SNP (7.3 × 10^−7^ mol/L) (S—yellow) and 5-[Mn^II^]^4.6^@CSNP (8.7 × 10^−7^ mol/L) (CS—black). sus: suspension; sup: supernatant; fil: filtrate. The exponent indicates the complex load in the nanoparticle.

The study of the luminescence of the materials possessing the manganese compounds was not easy. Indeed, fluorescence is a very sensitive phenomenon; multiple effects, known as luminescence quenching, can modify it or extinguish it partially or completely. The main way to achieve it in this study was by comparing between the material in suspension and the same solution stripped of the nanoparticles. We started with the [Mn^II^]@NP, and two methods were used. The first was the centrifugation of the suspension (4000 rpm, 10 min) in order to collect and study the supernatant without the particles. The second was the filtration of the suspension through 40 nm nylon filters to the same end. Emission spectra of these supernatants were collected for the most important load of each [Mn^II^]@NP type, with a concentration of 25 mg/L of particles in ethanol. Spectra are displayed in Figure 8 and Figure 9, showing the value of the integer for each curve (between 380 and 550 nm).

Figure 8a shows that after the filtration, the intensity recorded was slightly superior to what is observed for 5-[Mn^II^]@RSNP in suspension (0.7%) but both are very low compared to the other materials. As a consequence, the luminescence of the compound was completely quenched inside the particles and the emission observed in suspension likely comes from free [Mn^II^] compounds. As the intensity of this emission was particularly low, most compounds remained inside the particles. Considering the physical and chemical properties of the hybrid material, this was not very surprising. Indeed, resol is an inflatable and flexible material, so it makes sense that it would be able to prevent [Mn^II^] from leaking by mechanically obstructing the channels. On the other hand, the resol structure, composed of polyphenol, could provide a better link than silica or pure carbon, thanks to π-stacking or other interactions with the aromatic rings of the complex. We already demonstrated in a previous study that functionalisation of mesoporous silica with imidazoline or pyridine prevent dinuclear Mn^III^ compounds with pyridine or phenanthroline ligands from leaking [43,52]. And the same properties that make the resol network a good [Mn^II^] trap are also likely to quench its emission. The more interaction the fluorophores have with their environment, the more likely they are to transfer their energy to it and possibly de-excite in a non-radiative way. The emission of the supernatant after centrifugation was slightly more emissive (5.5%). This again suggests that the bulk of the emission came from free [Mn^II^] complexes released from the particle during the centrifugation. It makes sense that [Mn^II^] can be detached during a process that imposes stress on the particle rather than the filtration that is gentler for the material.

For 5-[Mn^II^]@SNP and 5-[Mn^II^]@CSNP, the observation was similar. However, a significant increase in the intensity already appeared with the filtration (17% and 130% respectively). Considering the nature of these materials, it seems logical that [Mn^II^] was more loosely bonded to it than to a resol-silica matrix. Unfortunately, this prevented us from assuming that [Mn^II^] luminescence was totally quenched inside the particle. 

For SNP and CSNP, it could be argued that the treatments applied to the particles were responsible for the [Mn^II^] release. The fact that [Mn^II^]@NP were brightly emissive but that filtration and centrifugation likely emptied the particles can be dismissed because the overall emission was less intense when it came to [Mn^II^]@NP suspensions than their counterpart in ethanol solutions (Figure S21). So [Mn^II^] was at least partially quenched inside the nanoparticles. Even in the case where the compound was not intact *(vide supra*), if anthracene carboxylate ligands were to be released, the intensity of their emission should be more important than what was observed.

Table 2 sums up in a more quantitative way the difference between the emission of free [Mn^II^] in solution, [Mn^II^] inside the nanoparticles and released [Mn^II^] with the two treatments. This table is only a guide line for SNP and CSNP, as it is hard to be sure that these nano-vectors totally quench [Mn^II^] emission. However, assuming that the integral of the intensity is proportional to [Mn^II^] concentration in solution, these results give a good approximation of the amount of complex released by RSNP in solution in different conditions, as [Mn^II^] was, very likely, completely quenched in this type of nanoparticle.

The emission of various [Mn^II^] loads for each nanoparticle type was studied (Figure S22). The emission became more intense when the particle’s load increased, suggesting that the quantity of [Mn^II^] released was in relation with the nanoparticle load. The signal emitted by [Mn^II^]@RSNP was, however, rather low for the detector’s settings. At the lowest concentration (2.3 × 10^−7^ mol/L), the bands other than the one at 412 nm seemed to disappear completely. In order to estimate if there was a simple relation between the particle load and the emission observed, a fit of the integrated normalised emission intensity for each particle type was performed (Figure S23). As the number of points is very limited, it was only a guide line, but the area described by the emission curve seemed proportional to the amount of [Mn^II^] loaded in the particle. This linear fit shows that for the particles that are believed to release [Mn^II^] more easily (CSNP and SNP) the slope was steeper than for the best complex trap (RSNP), which was not surprising. Finally, a deformation of the band at 462 nm was observed for some samples (Figure S24). It was attributed to an excimer luminescence due to [Mn^II^] released from the nanoparticles forming local aggregates.

All in all, the fluorescence observed in [Mn^II^]@NP suspension was mainly coming from the [Mn^II^] released in solution. [Mn^II^] luminescence was at least partially quenched inside the particles. From the difference in intensity observed between free [Mn^II^] in ethanol and the spectra of [Mn^II^]@NP, a significant amount of the complex still seems to be entrapped in the particles when they were redispersed in ethanol. RSNP appear to be the most suitable nano-vectors, as they do not instantly release [Mn^II^] in solution and they seem to be more resistant to treatments like centrifugation or filtration. CSNP are second, in terms of [Mn^II^] trapping, but are prone to leak a more important number of complexes. Finally, SNP appear to be the worse nanoparticles at retaining the complex inside, but seem to resist more than CSNP and less than RSNP when submitted to the above-mentioned treatments. The quantity of complexes that leak out of the particles appears to be somewhat proportional to the particle’s load, with a proportionality factor linked to the NP material type. Since the best retaining phase was apparently the resol one, we prepared the pure resol nanoparticles by basic treatment of the hybrid resol-silica nanoparticles [23]. Unfortunately, the porous structure of the pure resol phase collapsed, precluding any further utility for our purpose.

**Figure 10:**
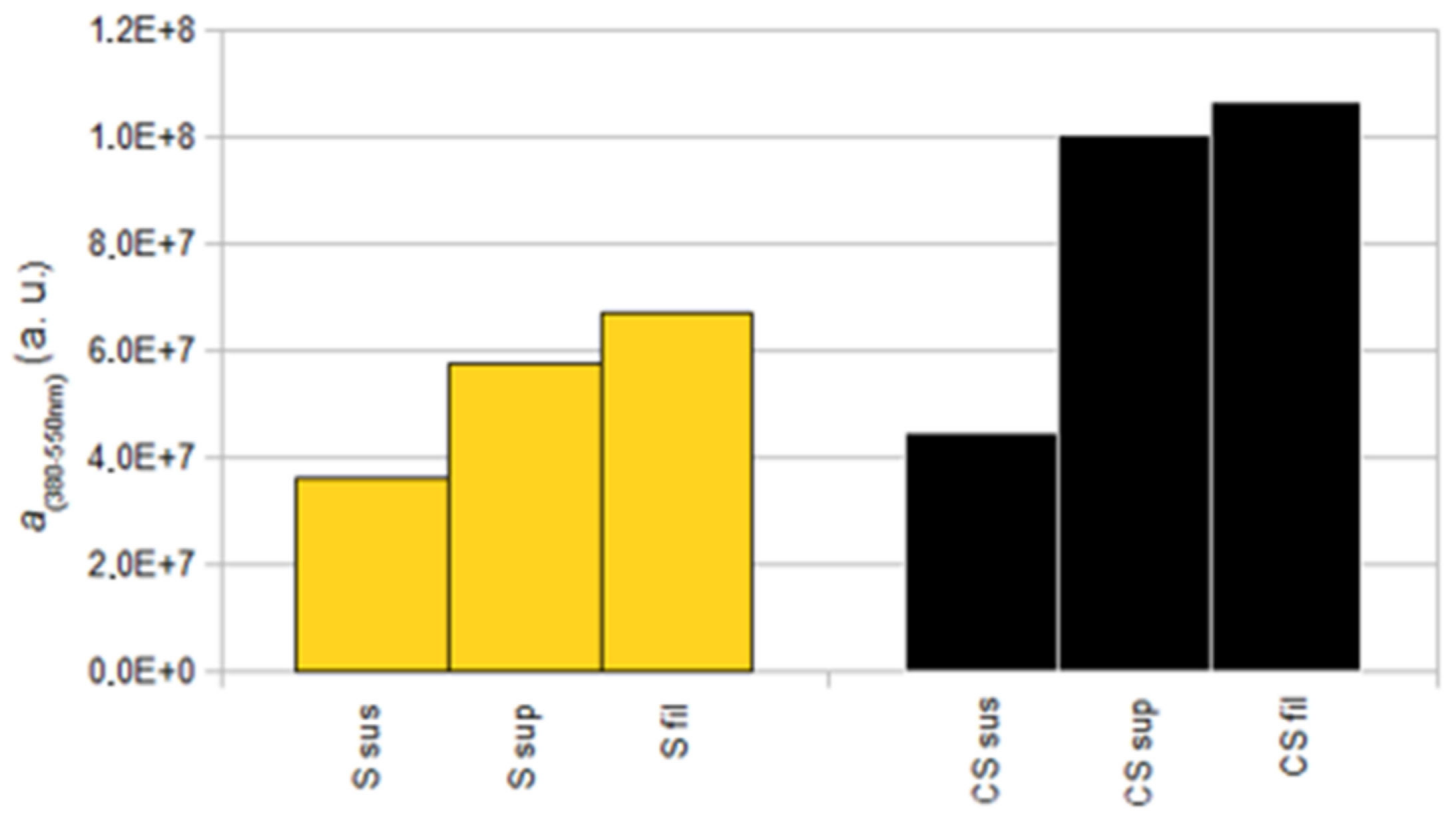
Value of the area below the emission curve between 380-550 nm for 5-[Mn^III^]^4.6^@SNP (4.6 × 10^−7^ mol/L) (S—yellow) and 5-[Mn^III^]^4.2^@CSNP (5.1 × 10^−7^ mol/L) (CS—black). sus: suspension; sup: supernatant; fil: filtrate.

In the case of [Mn^III^]@NP materials, the same protocol was applied, i.e., the luminescence of the compounds in suspension and the emission spectra of the solution with the nanoparticles was compared. The same treatment of the samples was followed as for [Mn^II^]@NP: filtration through a 40 nm nylon filter and centrifugation 10 min at 4000 rpm. Overall, the emission spectra of these materials were more difficult to interpret because the Mn^III^ complex was much less soluble in ethanol than the Mn^II^ compound and formed excitons at much lower concentration, as discussed before. All these measurements were recorded with the most important loads of [Mn^III^] and the emission of the nano-vectors was considered to be quenched, since a minimum amount of compound appeared to be sufficient to quench the luminescence of the nano-vector, as already observed for the materials containing the Mn^II^ complexes (Figure 10 and Figure S25). [Mn^III^]@SNP and [Mn^III^]@CSNP spectra can essentially be interpreted as the particles likely contributed to quenching the luminescence of the complex. When the spectra of the filtrate or the supernatant was recorded, the intensity of the emission increased because some more [Mn^III^] leaked out in solution during the process. A change in the intensity ratio between the bands at 412 nm and 462 nm was observed because the complexes extracted from the particles, which contributed to enhance the emission, were believed to make aggregates that contribute to the exciton bands. The method used to collect the solution seems, however, to have less impact on the shape and intensity of the spectra, which means that [Mn^III^] complexes were probably more loosely bonded to the particles than [Mn^II^]. The interpretation was not straightforward for [Mn^III^]@RSNP. The emission curves of [Mn^III^]@NP materials were recorded for the same nanoparticle concentration in ethanol (250 mg/L), but different [Mn^III^] loads (Figure S26). A large and intense band appeared at 470 nm for the most concentrated samples (5-[Mn^II^]^4.6^@RSNP and 3-[Mn^II^]^2.7^@RSNP) but not for the least-loaded one (1-[Mn^II^]^1.0^@RSNP). At the same time, this band was observed in several supernatants (Figure S25). As the concentration of the suspension prepared was lower (5.5 × 10^−7^ mol/L) than the one where this phenomenon was observed for free [Mn^III^ ] in ethanol solution (6.6 × 10^−6^ mol/L), we suggest that this band can be related to the luminescence of [Mn^III^] aggregates detached from particles, as previously observed. The fact that this deformation was more systematic and that we did not manage to mitigate it with different suspensions’ preparations was in accordance with the limited solubility of [Mn^III^] in ethanol. This hypothesis was strengthened by looking at the clean shape of the signal of the 5-[Mn^III^]^4.6^@RSNP filtrate, without a large 470 nm band. If [Mn^III^] forms large aggregates when released in solution, these aggregates could be removed more efficiently with a filter rather than centrifugation if they are stable.

Because of this intense deformation of the signal, it was not possible to represent the loss of the intensity more quantitatively for [Mn^III^]@RSNP. However, from the general shape of the signal and its modification upon filtration and centrifugation treatments, it is possible to state that [Mn^III^] seems less strongly bonded to RSNP than [Mn^II^]. Unfortunately, it could not be stated that the luminescence of [Mn^III^] was completely quenched for sure inside the particle, as the signal of the suspension and its supernatant differs. Another evidence that the [Mn^III^] complex was more prone to leak from RSNP than [Mn^II^] was deduced by comparing the total emission of the suspension to the signal of [Mn^III^]@SNP and [Mn^III^]@CSNP: it was much stronger than for [Mn^II^]@RSNP when compared to [Mn^II^]@SNP and [Mn^II^]@CSNP.

Figure 10 and Table 3 present the evolution of the integrated emission curves of [Mn^III^]@SNP and [Mn^III^]@CSNP. We chose not to represent [Mn^III^]@RSNP, as the distortion of the 470 nm band was too important, which would underestimate the leak in the calculation. With the same approach, as the signal was more deformed for supernatant and filtrate for [Mn^III^]@SNP and [Mn^III^]@CSNP, the leak was overestimated. Again, these numbers have to be largely nuanced, as it cannot be ascertained that [Mn^III^] was completely quenched inside the nanoparticles, which could again overestimate the leak.

On the other hand, linear fits between the integration value of the emission curves and the particle’s load were determined (Figure S27). From these results we can see that the measured intensity was proportional to the particle’s load. The only point that really stands out from this trend was sample 3-[Mn^III^]^1.2^@CSNP (1.5 × 10^−7^ mol/L), whose load seems to have been underestimated from the elemental analyses (1.2 wt% of complex *versus* a 3 wt% goal during the preparation). It has to be remembered that what the fluorimeter records were mainly (but not totally) the emission of free fluorophores in solution. So, these curves principally showed that the release rate of the fluorophores was the same for any complex load. Interestingly, we can see that in this case, the slope of [Mn^III^]@RSNP did not stand out compared to the other nanoparticles’ types, contrary to [Mn^II^] series (Figures S23 and S27) and in accordance with the leaking test. This result has however to be nuanced as, [Mn^III^]@RSNP, contrary to the two other nano-vectors, exhibited the strong excimer band at 470 nm, which makes the direct comparison difficult.

**Figure 11:**
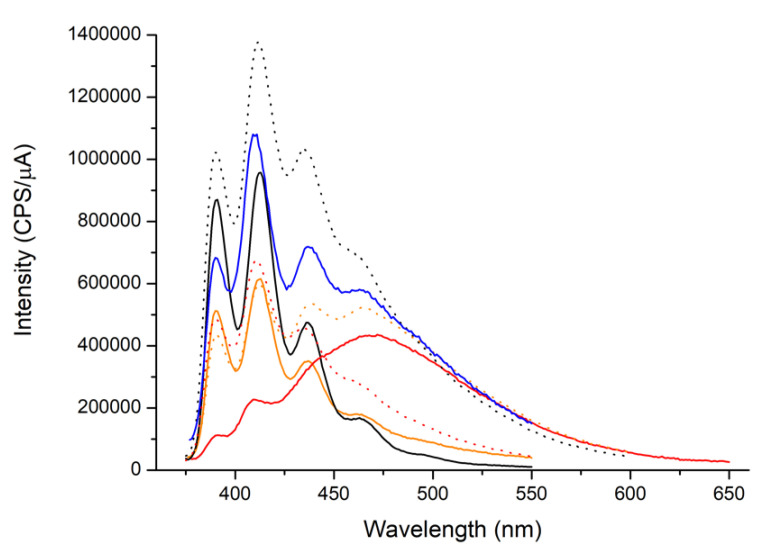
Emission of free [Mn^III^] (5.0 × 10^−7^ mol/L) (blue line), 5-[Mn^III^]^4.6^@RSNP (5.5 × 10^−7^ mol/L) (red line), 5-[Mn^III^]^3.8^@SNP (4.6 × 10^−7^ mol/L) (orange line) and 5-[Mn^III^]^4.2^@CSNP (5.0 × 10^−7^ mol/L) (black line). Wavelength of excitation: 362 nm. The emission spectra of the corresponding filtrates are represented in dotted lines.

The emission spectra measured for suspensions of [Mn^III^]@NP and free [Mn^III^] in ethanol for close concentrations are depicted in Figure 11. The overall emission of the compounds was much closer in terms of intensity to the free [Mn^III^] than what was observed for [Mn^II^] and [Mn^II^]@NP. This means that the majority of the compound was released in solution. And if we compare the intensity of free [Mn^III^] in solution to the one of the 5-[Mn^III^]4.2@CSNP filtrate, it was even more intense. This could be caused by damaged complexes whose free carboxylate moieties are likely more emissive than the coordinated ones.

To sum up, the interpretation of the luminescent properties of [Mn^III^]@NP was not straightforward for numerous reasons. The tetranuclear Mn^III^ compound is not very soluble in ethanol, quickly giving rise to exciton band emission that makes signal integration harder to compare. This compound had tendency to form aggregates that could have been carried away with particles during the purification processes. Nevertheless, what can be interpreted from these measurements is that [Mn^III^] was, overall, not as strongly bonded to the particles as [Mn^II^] could be. In addition, it seems that the [Mn^III^] signal was not totally quenched by the particle’s environment. This is in accordance with the chemical nature of the nano-vehicle. If the chemical environment in the nano-vehicle is less favourable to the complex stabilisation, it is also less likely to interact with its excited states and thus less prone to quench [Mn^III^] luminescence. Finally, the proportional relation between the particles’ theoretical load and the luminescence observed in suspension, also suggests that a significant amount of compound was likely to be intact, as it should have been far more intense and less deformed if the carboxylate had broken free.

## 4. Conclusions

A series of mesoporous nanoparticles based on hybrid resol-silica nanoparticles (RSNP) were synthesized. Thermal treatments yielded silica nanoparticles (SNP) through calcination under air and carbon-silica nanoparticles (CSNP) through carbonisation under N_2_ atmosphere. All these nanoparticles have the same average diameter (150 nm) and present a dendritic porosity but have different available volumes and mechanical properties. 

These nano-vehicles were used as support for the incorporation of a Mn^II^ dinuclear complex and a tetranuclear Mn^III^ compound presenting a butterfly core. In most cases, the insertion of the Mn compounds proved to be efficient (72% to 96% incorporation ratio). However, it appears that the Mn^III^ tetranuclear complexes tended to break into smaller dinuclear units when they entered the particles. In particular, the porosity of RSNP was modified when the compounds were inserted, probably due to the flexibility of the resol polymer network.

The magnetic measurements confirmed that both [Mn^II^] and [Mn^III^] complexes are present as dinuclear entities when incorporated inside the particles. These units display weak antiferromagnetic coupling and strong distortions of the octahedral environment of Mn ions. The study of the luminescence of the Mn compounds shows that they are both strongly fluorescent in the 390–462 nm range with a structured emission characteristic of anthracene derivatives. The shape of the emission spectra of [Mn^III^] is dependent on its concentration in ethanol and can display a large exciton band in the 460–470 nm range.

All three types of nano-vehicles are luminescent, with a large band at 407 nm. This band originates from the silica phase but its exact cause is difficult to ascertain. The incorporation of the manganese compounds inside the nano-vectors partially quenched the luminescence of both parts (metal complex and nano-vehicle). The study of the fluorescence of the nanoparticles’ suspensions and the supernatants showed that [Mn^II^] compounds seem to be more retained inside the particles than [Mn^III^]. However, all the material types, with the exception of [Mn^II^]@RSNP, proved to leak substantially. As a consequence, [Mn^II^]@RSNP appears to be the best suited material for theragnostic applications. Current work is in progress to further functionalize the nanoparticles with organic functions in order to better retain the metal complexes. 

## Figures and Tables

**Table 1:** Estimated manganese complex load (wt%) for the different nanoparticles from chemical analyses, TGA and magnetic measurements.

Material	wt% [Mn^II^]	Material	wt% [Mn^III^]
1-[Mn^II^]@RSNP	0.6	1-[Mn^III^]@RSNP	1.0
3-[Mn^II^]@RSNP	2.0	3-[Mn^III^]@RSNP	2.7
5-[Mn^II^]@RSNP	2.9	5-[Mn^III^]@RSNP	4.6
1-[Mn^II^]@CSNP	0.4	1-[Mn^III^]@CSNP	0.9
3-[Mn^II^]@CSNP	2.9	3-[Mn^III^]@CSNP	1.2
5-[Mn^II^]@CSNP	4.6	5-[Mn^III^]@CSNP	4.2
1-[Mn^II^]@SNP	1.2	1-[Mn^III^]@SNP	0.9
3-[Mn^II^]@SNP	2.9	3-[Mn^III^]@SNP	3.5
5-[Mn^II^]@SNP	3.9	5-[Mn^III^]@SNP	3.8

**Table 2:** Evolution of the integrate of the emitted intensity measured for all nanoparticle’s types loaded with Mn^II^ complexes.

Material	Capture ^1^	Centrifugation Release ^2^	Filtration Release ^3^
5-[Mn^II^]@RSNP	88	5.8	0.7
5-[Mn^II^]@SNP	38	51	17
5-[Mn^II^]@RSNP	46	138	130

^1^$\frac{a_{\mathrm{sol}}-a_{\mathrm{sus}}}{a_{\mathrm{sol}}}$ (%); ^2^ $\frac{\left|a_{\mathrm{sus}}-a_{\sup}\right|}{a_{\mathrm{sus}}}$ (%); ^3^ $\frac{\left|a_{\mathrm{sus}}-a_{\mathrm{fil}}\right|}{a_{\mathrm{sus}}}$ (%); a_sol_: area below the emission curve (380–550 nm) of free [Mn^II^] in ethanol suspension; a_sus_: area below the emission curve of [Mn^II^]@NP in ethanol suspension; a_sup_: area below the emission curve of the corresponding centrifugation supernatant; a_fil_: area below the emission curve of the corresponding filtrate.

**Table 3:** Evolution of the integrate of the emitted intensity measured for all types of nanoparticles loaded with Mn^III^ complexes.

Material	Capture ^1^	Centrifugation Release ^2^	Filtration Release ^3^
5-[Mn^III^]@SNP	47	87	59
5-[Mn^III^]@CSNP	40	139	125

^1^$\frac{a_{\mathrm{sol}}-a_{\mathrm{sus}}}{a_{\mathrm{sol}}}$ (%); ^2^ $\frac{\left|a_{\mathrm{sol}}-a_{\mathrm{sus}}\right|}{a_{\mathrm{sus}}}$ (%); ^3^ $\frac{\left|a_{\mathrm{sus}}-a_{\mathrm{fil}}\right|}{a_{\mathrm{sus}}}$ (%); a_sol_: area below the emission curve (380–550 nm) of free [Mn^III^] in ethanol suspension; a_sus_: area below the emission curve of [Mn^III^]@NP in ethanol suspension; a_sup_: area below the emission curve of the corresponding centrifugation supernatant; a_fil_: area below the emission curve of the corresponding filtrate.

## Data Availability

The data presented in this study are available on request from the corresponding author.

## Supplementary Materials

The following are available online at https://www.mdpi.com/2079-4991/11/3/774/s1, Figure S1: Infrared spectra of the mesoporous nanoparticles, Figure S2: Infrared spectra of [Mn^III^]@RSNP and [Mn^II^]@CSNP, Figure S3: ^29^Si NMR and ^13^C NMR spectra of the mesoporous nanoparticles, Figure S4: TGA of the mesoporous nanoparticles, Figure S5: TGA curves of empty SNP nanoparticles and the ones loaded with the Mn^III^ complex, 1-[Mn^III^]@SNP, 3-[Mn^III^]@SNP and 5-[Mn^III^]@SNP, Figure S6: TGA curves of empty SNP nanoparticles and the ones loaded with the Mn^II^ complex, 1-[Mn^II^]@SNP, 3-[Mn^II^]@SNP and 5-[Mn^II^]@SNP, Figure S7: TGA curves of empty CSNP nanoparticles and the ones loaded with the Mn^II^ complex, 1-[Mn^II^]@CSNP, 3-[Mn^II^]@CSNP and 5-[Mn^II^]@CSNP, Figure S8: SEM images of RSNP, SNP and CSNP, Figure S9 TEM images of 5-[Mn^III^]@SNP, Figure S10: TEM images of 5-[Mn^III^]@CSNP, Figure S11: N_2_-sorption isotherms of extracted RSNP, calcined SNP and carbonised CSNP, Figure S12: N_2_-sorption isotherms of 5-[Mn^III^]@RSNP, 3-[Mn^III^]@RSNP and 1-[Mn^III^]@RSNP, Figure S13: N_2_-sorption isotherms of 5-[Mn^II^]@RSNP, 3-[Mn^II^]@RSNP and 1-[Mn^II^]@RSNP, Figure S14: N_2_-sorption isotherms of 5-[Mn^III^]@SNP, 3-[Mn^III^]@SNP and 1-[Mn^III^]@SNP, Figure S15: N_2_-sorption isotherms of 5-[Mn^II^]@SNP, 3-[Mn^II^]@SNP and 1-[Mn^II^]@SNP, Figure S16: N_2_-sorption isotherms of 5-[Mn^III^]@CSNP, 3-[Mn^III^]@CSNP and 1-[Mn^III^]@CSNP, Figure S17: N_2_-sorption isotherms of 5-[Mn^II^]@CSNP, 3-[Mn^II^]@CSNP and 1-[Mn^II^]@CSNP, Figure S18: χ_M_T vs. T for RSNP SNP and CSNP and X-band EPR spectrum of CSNP, Figure S19. χ_M_T vs. T for [Mn^II^]@RSNP and [Mn^III^]@RSNP and the corresponding simulated curves, Figure S20: Emission spectra of [Mn]@NP with the smallest Mn compound load for each NP type compared to the emission spectra of empty NP, Figure S21: Emission spectra of free [Mn^II^], [Mn^II^]@RSNP, [Mn^II^]@SNP and [Mn^II^] CSNP, Figure S22: Emission spectra of [Mn^II^]@NP for each NP type, Figure S23: Area below the emission curve versus particle’s load for [Mn^II^] of [Mn^II^]@RSNP, [Mn^II^]@SNP and [Mn^II^]@CSNP suspensions and the corresponding fits with their equations, Figure S24: Emission spectra of [Mn^II^]@RSNP, Figure S25: Emission spectra of [Mn^III^]@NP, Figure S26: Emission spectra of [Mn^III^]@NP, Figure S27: Area below the emission curve versus particle’s load of [Mn^III^]@RSNP, [Mn^III^]@SNP and [Mn^III^]@CSNP suspensions and the corresponding fits with their equations, Table S1: Porosity data from nitrogen sorption isotherms for RSNP, SNP and CSNP, Table S2: Porosity data from N_2_-sorption isotherms for [Mn^III^]@RSNP, Table S3: Porosity data from N_2_-sorption isotherms for [Mn^II^]@RSNP, Table S4: Porosity data from N_2_-sorption isotherms for [Mn^III^]@SNP; Table S5: Porosity data from N_2_-sorption isotherms for [Mn^II^]@SNP, Table S6: Porosity data from N_2_-sorption isotherms for [Mn^III^]@CSNP, Table S7: Porosity data from N_2_-sorption isotherms for [Mn^II^]@CSNP.
