# Supplementary material for: Incorporation of Manganese Complexes within Hybrid Resol-Silica and Carbon-Silica Nanoparticles
Source: Nanomaterials (Basel). 2021 Mar 18;11(3):774. doi: 10.3390/nano11030774 (PMC8002901; doi:10.3390/nano11030774)
Supplement: Supplementary file 1 [file nanomaterials-11-00774-s001.pdf]

Article

Electronic Supporting Information

Incorporation of manganese complexes within hybrid resol-silica and carbon-silica nanoparticles

François-Xavier Turquet [1,2], Montserrat Corbella [2,*], Clémentine Fellah [3], Gilles Montagnac [3], Bruno Reynard [3], Laurent Bonneviot [1], Kun Zhang [4], and Belén Albela [1,*]

[1] Laboratoire de Chimie, Ecole Normale Supérieure de Lyon; Université de Lyon, 46 Allée d'Italie, 69364 Lyon Cedex 07, France; e-Mails: fx.turquet@protonmail.com (F.-X.T.); laurent.bonneviot@ens-lyon.fr (L.B.) , belen.albela@ens-lyon.fr (B.A.)

[2] Departament de Química Inorgànica i Orgànica (Secció Inorgànica), Universitat de Barcelona, Martí I Franquès 1-11, 08028 Barcelona, Spain; e-Mail: montse.corbella@qi.ub.es (M.C.)

[3] Laboratoire de Géologie, Ecole Normale Supérieure de Lyon; Université de Lyon, 46 Allée d'Italie, 69364 Lyon Cedex 07, France; e-Mails: clementine.fellah@ens-lyon.fr (C.F.), gilles.montagnac@ens-lyon.fr (G.M.), bruno.reynard@ens-lyon.fr (B.R.)

[4] Shanghai Key Laboratory of Green Chemistry and Chemical Processes, Department of Chemistry, East China Normal University, 3663 North ZhongShan Rd., Shanghai 200062, China,; e-Mail: kzhang@chem.ecnu.edu.cn

Infrared Spectra

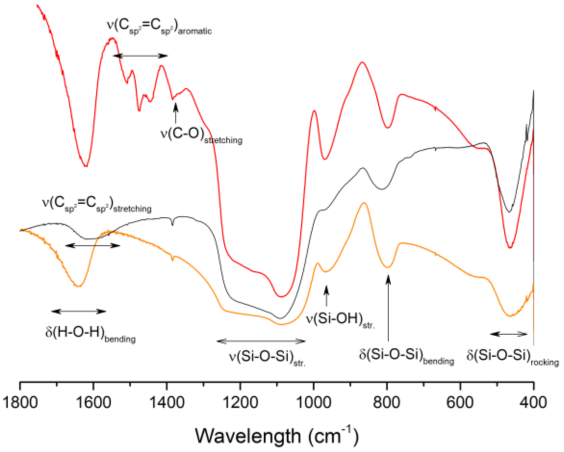

Figure S1. Infrared spectra of mesoporous silica nanoparticles (SNP) (orange line), mesoporous resole-silica nanoparticles (RSNP) (red line) and mesoporous carbon-silica nanoparticles (CSNP) (black line).

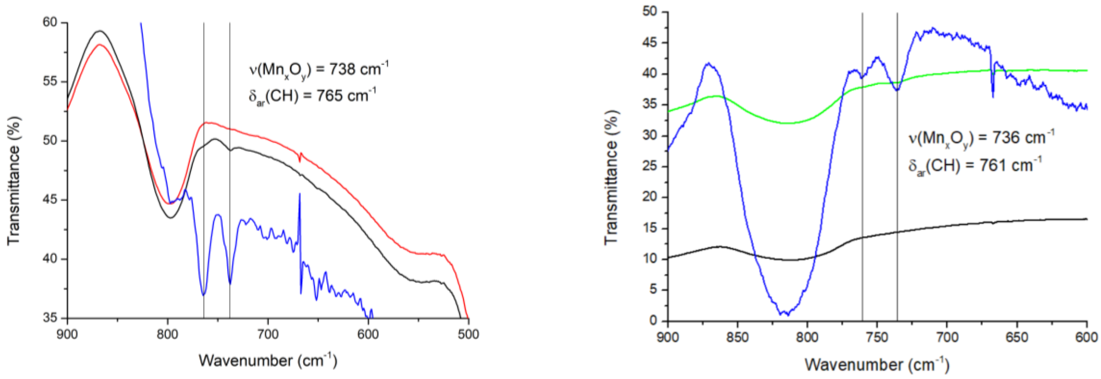

(**a**) (**b**)

Figure S2. Infrared spectra of [Mn^{III}]@RSNP and [Mn^{II}]@CSNP: (**a**) hybrid resol-silica nanoparticles (RSNP) (red line), [Mn^{III}]-loaded particles ([Mn^{III}]@RSNP) (black line) and difference between the two spectra (blue line); (**b**) carbon-silica nanoparticles (CSNP) (black line) and [Mn^{II}]-loaded particles [Mn^{II}]@CSNP (green line) and the difference between the two spectra (blue line).

NMR Spectra

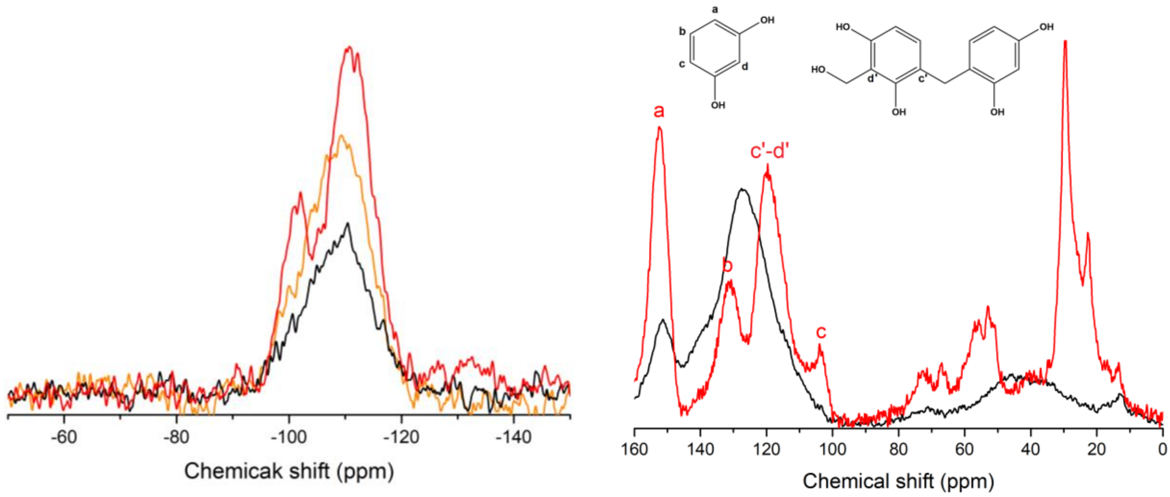

(**a**) (**b**)

Figure S3. (**a**) ^{29}Si NMR spectra of RSNP (red), SNPs (orange) and CSNP (black); (**b**) ^{13}C NMR spectra of RSNP (red) and CSNP (black), with matching carbon attributions.

Thermogravimetrical Analysis (TGA)

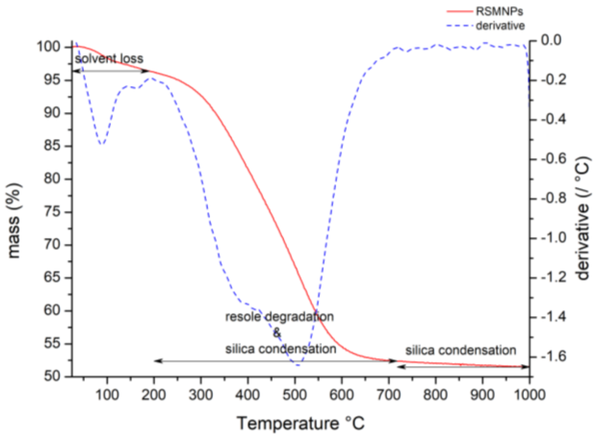

(a)

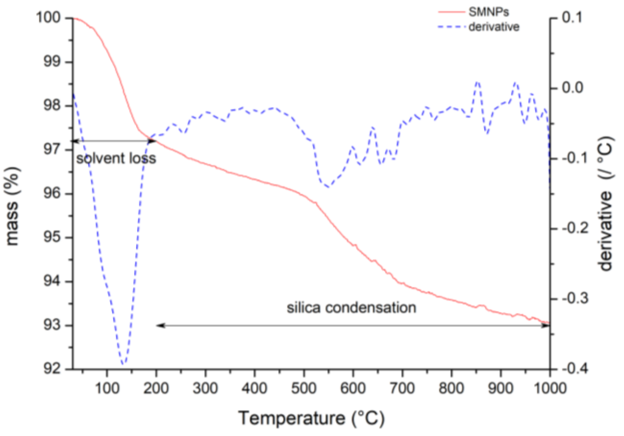

(b)

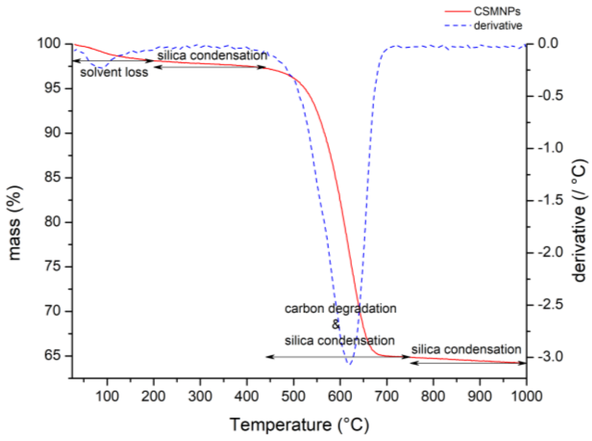

(c)

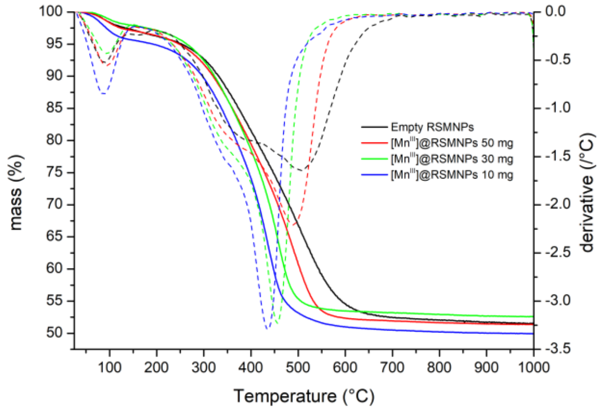

(d)

Figure S4. (**a**) TGA of mesoporous hybrid resol-silica nanoparticles (RSNP) (plain line) and its derivative (dashed line). It evidences a mass ratio of 43% silica and 55% resorcinol. (**b**) TGA of silica mesoporous nanoparticles (SNP) (plain line) and its derivative (dashed line). (**c**) TGA of hybrid carbonsilica mesoporous nanoparticles (CSNP) (plain line) and its derivative (dashed line). It shows a mass ratio of 64% silica and 33% carbon. (**d**) TGA curves of empty RSNP nanoparticles and the ones loaded with 10, 30 and 50 mg of [Mn^{III}] per gram of material, 1-[Mn^{III}]@RSNP, 3-[Mn^{III}]@RSNP and 5-[Mn^{III}]@RSNP, respectively. Plain line represents mass (%) per temperature (°C), dashed line its derivative.

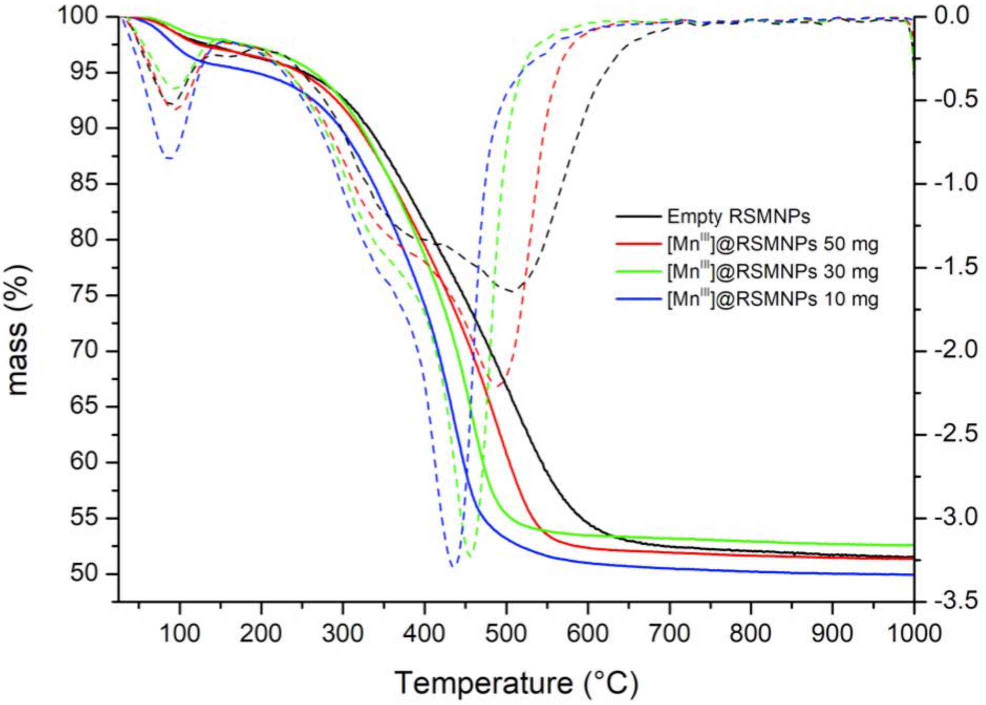

Figure S5. TGA curves of empty SNP nanoparticles and the ones loaded with 10, 30 and 50 mg of Mn^{III} complex per gram of material, 1-[Mn^{III}]@SNP (blue), 3-[Mn^{III}]@SNP (green) and 5-[Mn^{III}]@SNP (red), respectively. Plain line represents mass (%) per temperature (°C), dashed line its derivative.

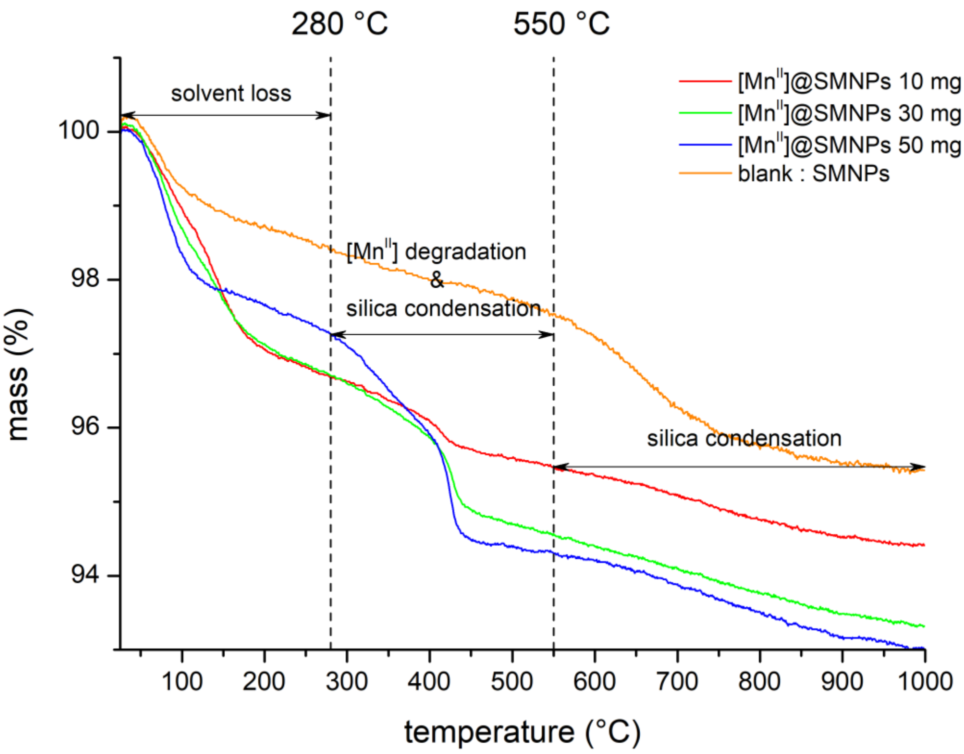

Figure S6. TGA curves of empty SNP nanoparticles (orange) and the ones loaded with 10, 30 and 50 mg of Mn^{II} complex per gram of material, 1-[Mn^{II}]@SNP (red), 3-[Mn^{II}]@SNP (green) and 5-[Mn^{II}]@SNP (blue), respectively.

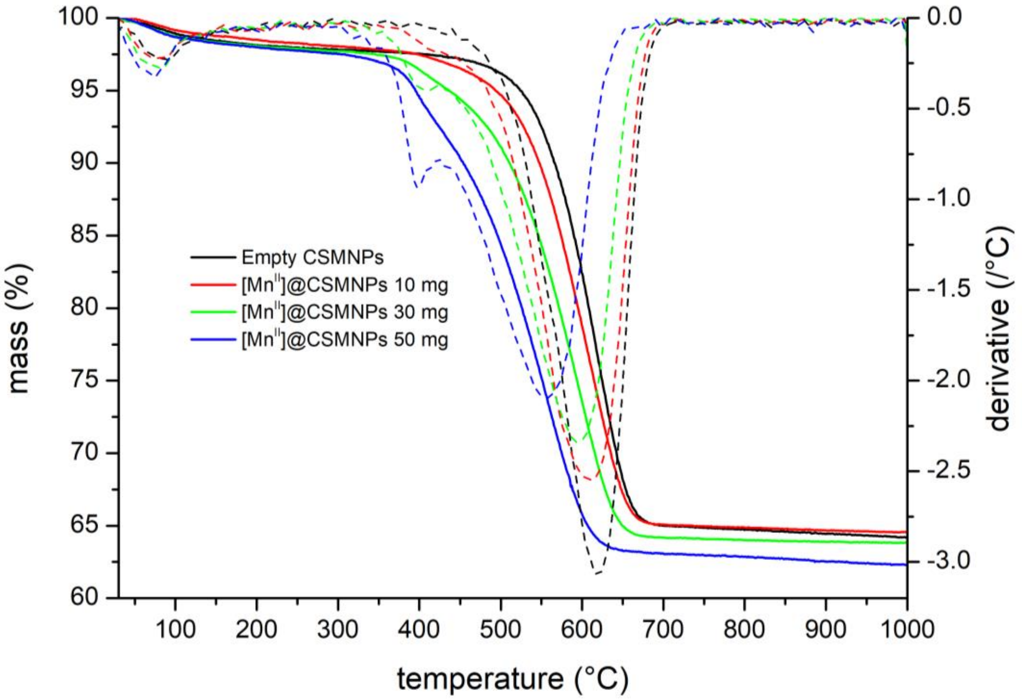

Figure S7. TGA curves of empty CSNP nanoparticles (black) and the ones loaded with 10, 30 and 50 mg of Mn^{II} complex per gram of material, 1-[Mn^{II}]@CSNP (red), 3-[Mn^{II}]@CSNP (green) and 5-[Mn^{II}]@CSNP (blue), respectively. Plain line represents mass (%) per temperature (°C), dashed line its derivative.

SEM and TEM Images

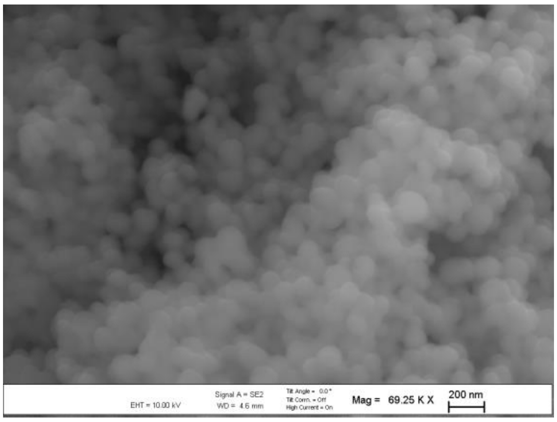

(**a**)

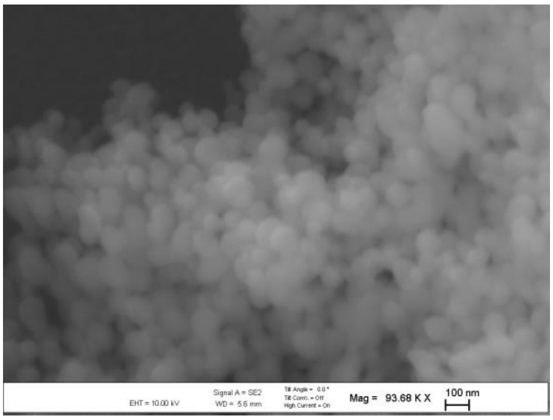

(**b**)

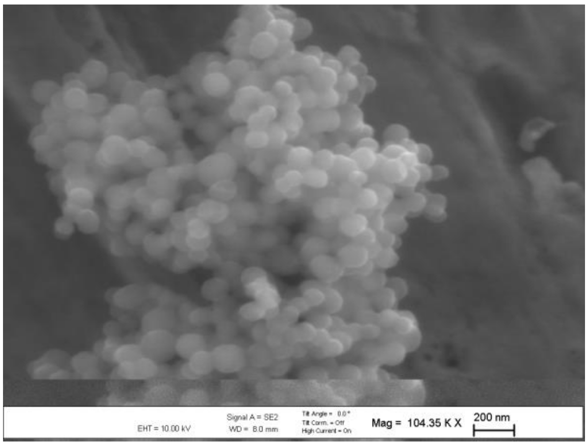

(c)

Figure S8. (**a**) SEM images of mesoporous hybrid resol-silica nanoparticles (RSNP), (**b**) mesoporous silica nanoparticles (SNP) and (**c**) hybrid carbon-silica nanoparticles (CSNP).

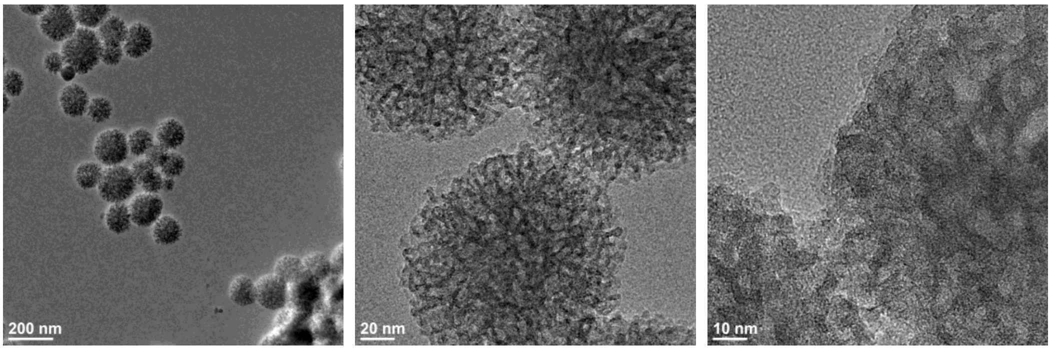

Figure S9. TEM images of 5-[Mn^{III}]@SNP.

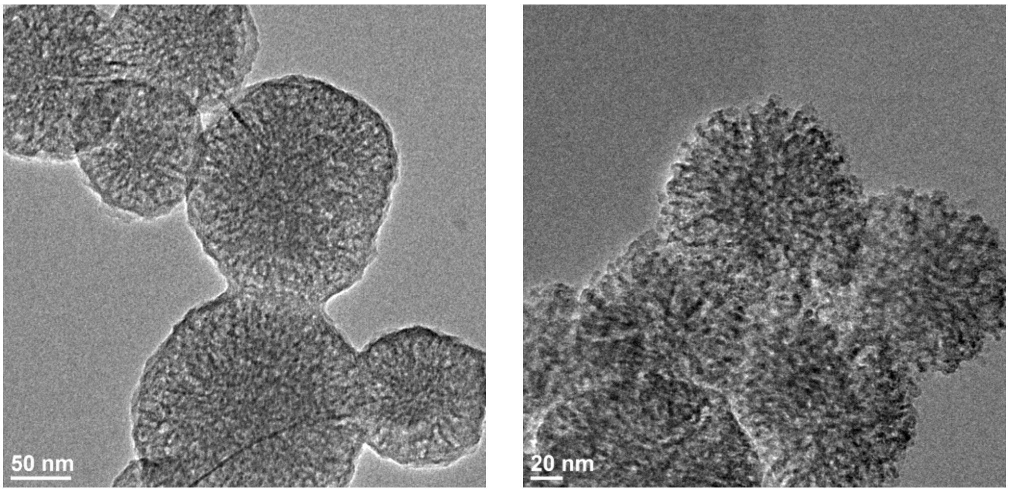

Figure S10. TEM images of 5-[Mn^{III}]@CSNP.

Nitrogen Sorption Isotherms

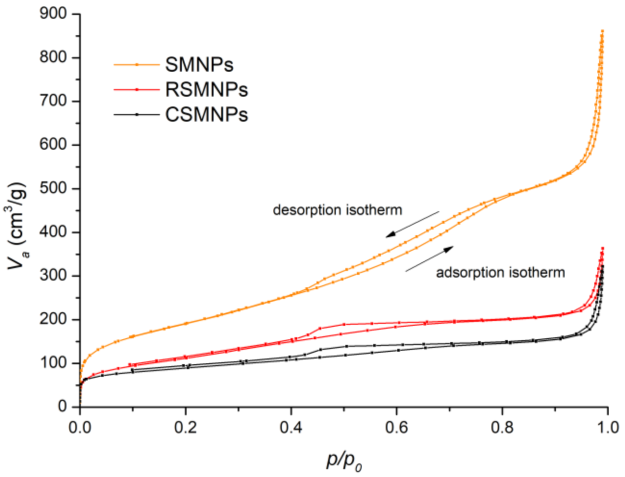

Figure S11. N_2-sorption isotherms at 77 K of extracted hybrid resol-silica nanoparticles (RSNP), calcined silica nanoparticles (SNP) and carbonised carbon-silica nanoparticles (CSNP).

Table S1. Porosity data from nitrogen sorption isotherms (77 K) for extracted hybrid silica-resol mesoporous nanoparticles (RSNP), calcined silica mesoporous nanoparticles (SNP) and carbonised mesoporous carbon-silica nanoparticles (CSNP).

	Sample		
	RSMNPs	SMNPs	CSMNPs
	BET		
V_t (cm^3/g) [a]	0.55	1.33	0.48
$a_{\mathrm{S,BET}}$ (m^2/g) [b]	404	689	313
$\mathrm{C}_{\mathrm{BET}}$	102	100	900
	t-plot		
a_{int} (m^2/g) [c]	367	654	206
a_{ext} (m^2/g) [d]	39	114	39
V_{micro} (cm^3/g) [c]	8.7×10^{-3}	7.3×10^{-3}	4.7×10^{-2}
V_{meso} (cm^3/g) [d]	0.27	0.65	0.19
V_{meso}/V_t	0.49	0.49	0.39
	BJH		
D_{BJH} (nm) [e]	2.5	2.5	2.4
D_{min} (nm) [f]	1.4	1.5	1.4
D_{max} (nm) [g]	4.2	7.1	4.8

From BET plot (a) for $p/p_0 = 0.99$; (b) $0.05 \geq p/p_0 \geq 0.16$. Calculated from t-plot (c) for $t \leq 0.5$; (d) for $t \geq 1.0$. From BJH plot, with $D_i = 2r_i$ (e) for $\frac{dV_p}{dr_p}_{\max}$; (f) for $\frac{dV_p}{dr_p}_{\max} - \left(\frac{dV_p}{dr_p}_{\max}\right)/2$; (g) for $\frac{dV_p}{dr_p}_{\max} + \left(\frac{dV_p}{dr_p}_{\max}\right)/2$.

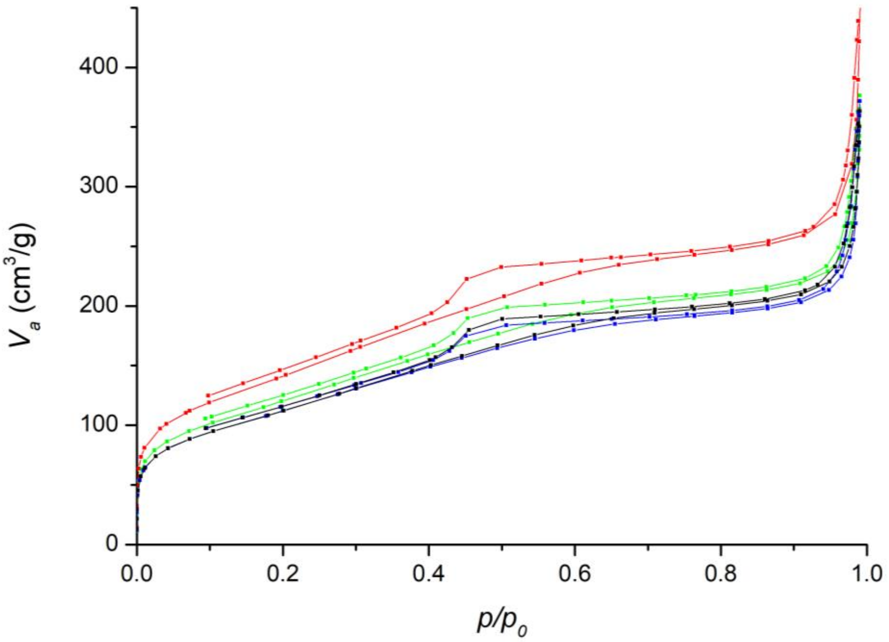

Figure S12. N_2-sorption isotherms at 77 K of [Mn^{III}]@RSNP loaded with 50 (blue), 30 (green) and 10 mg (red) of the Mn^{III} complex for 1.00 g of RSNP, 5-[Mn^{III}]@RSNP, 3-[Mn^{III}]@RSNP and 1-[Mn^{III}]@RSNP, respectively. Black line and points are the isotherms of the complex-free RSNP. Curves were corrected with results of the elemental analysis in order to reflect more accurately the characteristics per gram of support instead of full material.

Table S2. Porosity data from N_2-sorption isotherms for [Mn^{III}]@RSNP: BET: Total pore volume (V_t), BET area ($a_{S,BET}$) and C coefficient (C_{BET}); t-plot : internal (a_{int}) and external (a_{ext}) pore area and microporous (V_{meso}) and mesoporous (V_{meso}) volume; microporous ratio (V_{micro}/V_t) of silica nanoparticles. The head-column number for each sample is the [Mn^{III}] complex load deduced from material Mn elemental analysis, which corresponds to RSNP, 1-[Mn^{III}]@RSNP, 3-[Mn^{III}]@RSNP and 5-[Mn^{III}]@RSNP, respectively.

	Samples			
	0%	1.0%	2.7%	4.6%
	BET			
V_t (cm^3/g) [a]	0.55	0.67	0.57	0.55
$a_{\mathrm{S,BET}}$ (m^2/g) [b]	404	505	422	386
C_{BET}	102	103	102	95
	t-plot			
a_{int} (m^2/g) [c]	367	474	376	346
a_{ext} (m^2/g) [d]	39	50	39	37
V_{micro} (cm^3/g) [c]	8.7×10^{-3}	6.4×10^{-3}	1.2×10^{-2}	9.7×10^{-3}
V_{meso} (cm^3/g) [d]	0.27	0.33	0.28	0.25
V_{meso}/V_t	0.49	0.49	0.48	0.44
	BJH			
D_{BJH} (nm) [e]	2.5	2.5	2.5	2.5
D_{min} (nm) [f]	1.4	1.4	1.4	1.4
D_{max} (nm) [g]	4.2	3.7	3.7	3.7

From BET plot (a) for $p/p_0 = 0.99$; (b) $0.05 \geq p/p_0 \geq 0.16$. Calculated from t-plot (c) for $t \leq 0.5$; (d) for $t \geq 1.0$. From BJH plot, with $D_i = 2r_i$ (e) for $\frac{dV_p}{dr_p}_{\mathrm{max}}$; (f) for $\frac{dV_p}{dr_p}_{\mathrm{max}} - \left(\frac{dV_p}{dr_p}_{\mathrm{max}}\right)/2$; (g) for $\frac{dV_p}{dr_p}_{\mathrm{max}} + \left(\frac{dV_p}{dr_p}_{\mathrm{max}}\right)/2$.

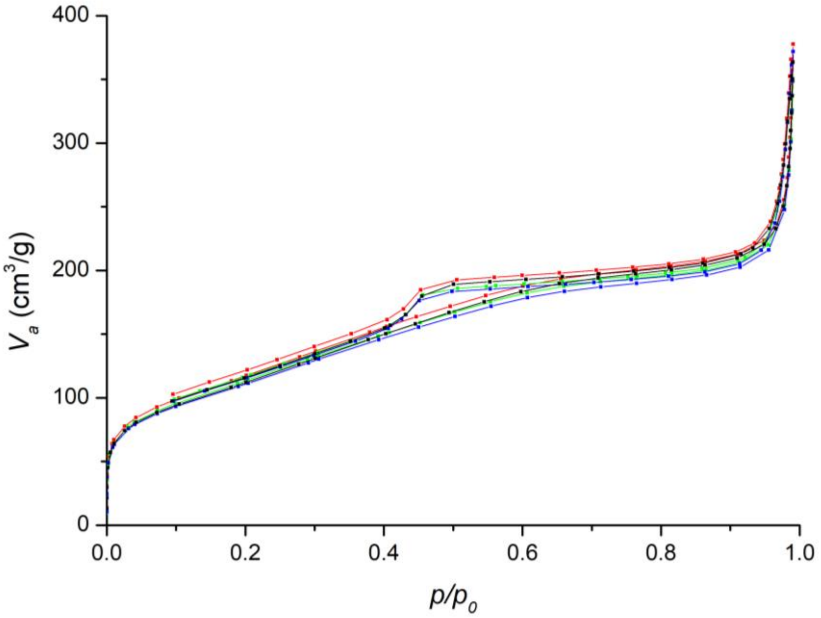

Figure S13. N_2-sorption isotherms at 77 K of [Mn^{II}]@RSNP loaded with 50 (blue), 30 (green) and 10 mg (red) of the Mn^{II} complex for 1.00 g of RSNP, 5-[Mn^{II}]@RSNP, 3-[Mn^{II}]@RSNP and 1-[Mn^{II}]@RSNP, respectively. Black line and points are the isotherms of the complex-free RSNP. Curves were corrected with results of the elemental analysis in order to reflect more accurately the characteristics per gram of support instead of full material.

Table S3. Porosity data from N_2-sorption isotherms for [Mn^{II}]@RSNP: BET: Total pore volume (V_t), BET area ($a_{S,BET}$) and C coefficient (C_{BET}); t-plot: internal (a_{int}) and external (a_{ext}) pore area and microporous (V_{meso}) and mesoporous (V_{meso}) volume; microporous ratio (V_{micro}/V_t) of silica nanoparticles. The head-column number for each sample is the [Mn^{II}] load deduced from material Mn elemental analysis, which corresponds to RSNP, 1-[Mn^{II}]@RSNP, 3-[Mn^{II}]@RSNP and 5-[Mn^{II}]@RSNP, respectively.

	Samples			
	0%	0.6%	2.0%	2.9%
	BET			
V_t (cm^3/g) [a]	0.55	0.57	0.52	0.54
$a_{\mathrm{S,BET}}$ (m^2/g) [b]	404	420	401	388
C_{BET}	102	103	102	98
	t-plot			
a_{int} (m^2/g) [c]	367	378	383	363
a_{ext} (m^2/g) [d]	39	39	41	38
V_{micro} (cm^3/g) [c]	8.7×10^{-3}	1.1×10^{-2}	3.1×10^{-3}	4.7×10^{-3}
V_{meso} (cm^3/g) [d]	0.27	0.27	0.26	0.25
V_{meso}/V_t	0.49	0.48	0.48	0.46
	BJH			
D_{BJH} (nm) [e]	2.5	2.5	2.5	2.4
D_{min} (nm) [f]	1.4	1.4	1.4	1.4
D_{max} (nm) [g]	4.2	4.2	7.1	3.7

From BET plot (a) for $p/p_0 = 0.99$; (b) $0.05 \geq p/p_0 \geq 0.16$. Calculated from t-plot (c) for $t \leq 0.5$; (d) for $t \geq 1.0$. From BJH plot, with $D_i = 2r_i$ (e) for $\frac{dV_p}{dr_p}_{\mathrm{max}}$; (f) for $\frac{dV_p}{dr_p}_{\mathrm{max}} - \left(\frac{dV_p}{dr_p}_{\mathrm{max}}\right)/2$; (g) for $\frac{dV_p}{dr_p}_{\mathrm{max}} + \left(\frac{dV_p}{dr_p}_{\mathrm{max}}\right)/2$.

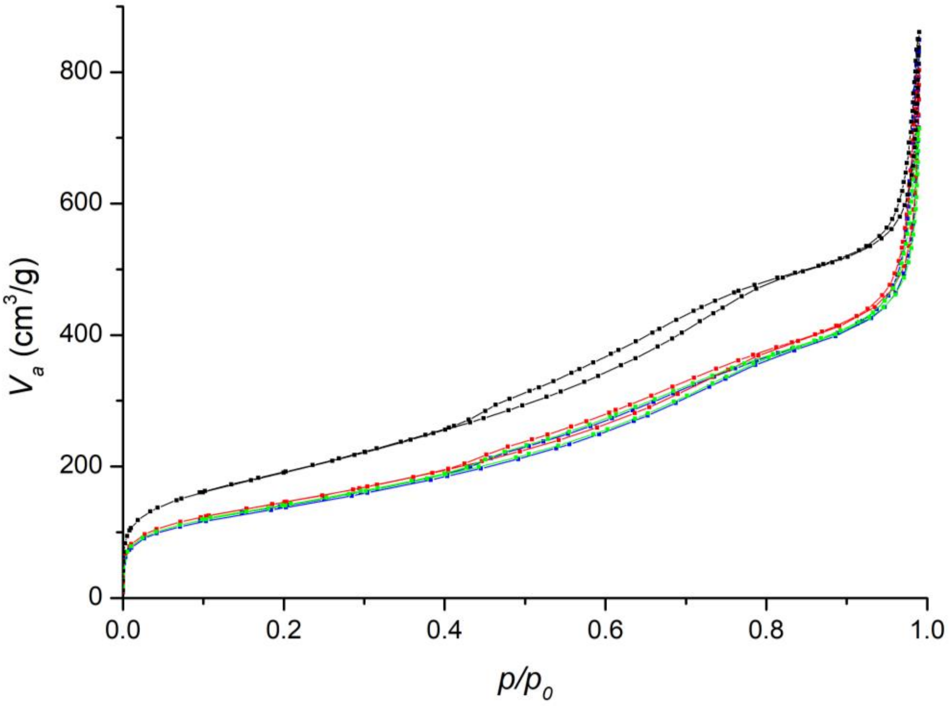

Figure S14. N_2-sorption isotherms at 77 K of [Mn^{III}]@SNP loaded with 50 (blue), 30 (green) and 10 mg (red) of the Mn^{III} complex for 1.00 g of SNPs, 5-[Mn^{III}]@SNP, 3-[Mn^{III}]@SNP and 1-[Mn^{III}]@SNP, respectively. Black line and points are the isotherms of the complex-free SNP. Curves were corrected with results of the elemental analysis in order to reflect more accurately the characteristics per gram of support instead of full material.

Table S4. Porosity data from N_2-sorption isotherms for [Mn^{III}]@SNP: BET : Total pore volume (V_t), BET area ($a_{S,BET}$) and C coefficient (C_{BET}); t-plot : internal (a_{int}) and external (a_{ext}) pore area and microporous (V_{meso}) and mesoporous (V_{meso}) volume; microporous ratio (V_{micro}/V_t) of silica nanoparticles. The head-column number for each sample is the [Mn^{III}] load deduced from material Mn elemental analysis, which corresponds to SNP, 1-[Mn^{III}]@SNP, 3-[Mn^{III}]@SNP and 5-[Mn^{III}]@SNP, respectively.

	Samples			
	0%	0.9%	2.5%	3.8%
	BET			
V_t (cm^3/g) [a]	1.33	1.22	1.07	1.24
$a_{S,BET}$ (m^2/g) [b]	689	520	494	477
C_{BET}	100	107	103	100
	t-plot			
a_{int} (m^2/g) [c]	654	504	471	462
a_{ext} (m^2/g) [d]	114	134	118	121
V_{micro} (cm^3/g) [c]	7.3×10^{-3}	2.0×10^{-3}	4.0×10^{-3}	7.3×10^{-4}
V_{meso} (cm^3/g) [d]	0.65	0.47	0.46	0.45
V_{meso}/V_t	0.49	0.38	0.42	0.35
	BJH			
D_{BJH} (nm) [e]	2.5	2.7	2.7	2.7
D_{min} (nm) [f]	1.5	1.5	1.5	1.5
D_{max} (nm) [g]	7.1	7.1	7.1	7.1

From BET plot (a) for $p/p_0 = 0.99$; (b) $0.05 \geq p/p_0 \geq 0.16$. Calculated from t-plot (c) for $t \leq 0.5$; (d) for $t \geq 1.0$. From BJH plot, with $D_i = 2r_i$ (e) for $\frac{dV_p}{dr_p}_{\max}$; (f) for $\frac{dV_p}{dr_p}_{\max} - \left(\frac{dV_p}{dr_p}_{\max}\right)/2$; (g) for $\frac{dV_p}{dr_p}_{\max} + \left(\frac{dV_p}{dr_p}_{\max}\right)/2$.

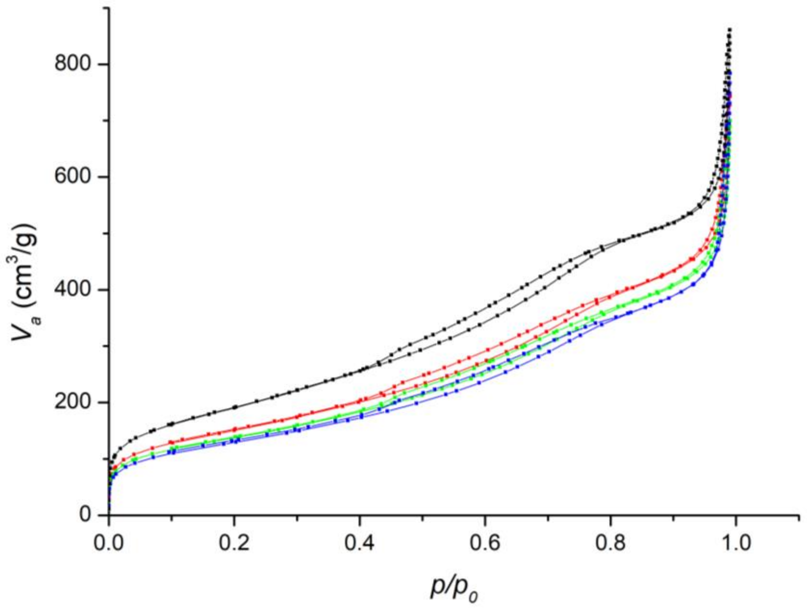

Figure S15. N_2-sorption isotherms at 77 K of [Mn^{II}]@SNP loaded with 50 (blue), 30 (green) and 10 mg (red) of the Mn^{II} complex for 1.00 g of SNP, 5-[Mn^{II}]@SNP, 3-[Mn^{II}]@SNP and 1-[Mn^{II}]@SNP, respectively. Black line and points are the isotherms of the complex-free SNP. Curves were corrected with results of the elemental analysis in order to reflect more accurately the characteristics per gram of support instead of full material.

Table S5. Porosity data from N_2-sorption isotherms for $[Mn^{II}]$@SNP: BET : Total pore volume (V_t), BET area ($a_{S,BET}$) and C coefficient (C_{BET}); t-plot : internal (a_{int}) and external (a_{ext}) pore area and microporous (V_{meso}) and mesoporous (V_{meso}) volume; microporous ratio (V_{micro}/V_t) of silica nanoparticles. The head-column number for each sample is the $[Mn^{II}]$ complex load deduced from material Mn elemental analysis, which corresponds to RSNP, 1-$[Mn^{II}]$@SNP, 3-$[Mn^{II}]$@SNP and -$[Mn^{II}]$@SNP, respectively.

	Samples			
	0%	1.2%	2.9%	3.9%
	BET			
V_t (cm^3/g) [a]	1.33	1.37	1.05	1.15
$a_{S,BET}$ (m^2/g) [b]	689	652	482	449
C_{BET}	100	131	107	100
	t-plot			
a_{int} (m^2/g) [c]	654	611	453	439
a_{ext} (m^2/g) [d]	114	156	118	107
V_{micro} (cm^3/g) [c]	7.3×10^{-3}	9.3×10^{-3}	6.9×10^{-3}	-
V_{meso} (cm^3/g) [d]	0.65	0.60	0.45	0.44
V_{meso}/V_t	0.49	0.52	0.42	0.37
	BJH			
D_{BJH} (nm) [e]	2.5	2.7	2.7	2.7
D_{min} (nm) [f]	1.5	1.5	1.5	1.5
D_{max} (nm) [g]	7.1	7.1	8.1	8.1

From BET plot (a) for $p/p_0 = 0.99$; (b) $0.05 \geq p/p_0 \geq 0.16$. Calculated from t-plot (c) for $t \leq 0.5$; (d) for $t \geq 1.0$. From BJH plot, with $D_i = 2r_i$ (e) for $\frac{dV_p}{dr_p}_{\max}$; (f) for $\frac{dV_p}{dr_p}_{\max} - \left(\frac{dV_p}{dr_p}_{\max}\right)/2$; (g) for $\frac{dV_p}{dr_p}_{\max} + \left(\frac{dV_p}{dr_p}_{\max}\right)/2$.

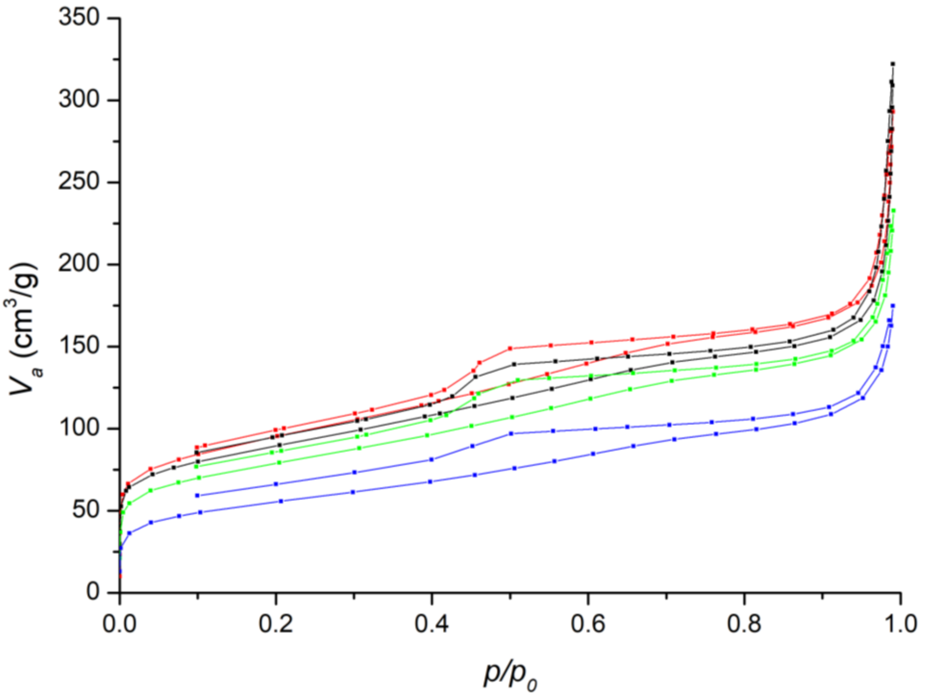

Figure S16. N_2-sorption isotherms at 77 K of [Mn^{III}]@CSNP loaded with 50 (blue), 30 (green) and 10 mg (red) of the Mn^{III} complex for 1.00 g of SNP, 5-[Mn^{III}]@CSNP, 3-[Mn^{III}]@CSNP and 1-[Mn^{III}]@CSNP, respectively. Black line and points are the isotherms of the complex-free CSNP. Curves were corrected with results of the elemental analysis in order to reflect more accurately the characteristics per gram of support instead of full material.

Table S6. Porosity data from N_2-sorption isotherms for [Mn^{III}]@CSNP: BET: Total pore volume (V_t), BET area ($a_{S,BET}$) and C coefficient (C_{BET}); t-plot : internal (a_{int}) and external (a_{ext}) pore area and microporous (V_{meso}) and mesoporous (V_{meso}) volume; microporous ratio (V_{micro}/V_t) of silica nanoparticles. The head-column number for each sample is the [Mn^{III}] load deduced from material Mn elemental analysis, which corresponds to CSNP, 1-[Mn^{III}]@CSNP, 3-[Mn^{III}]@CSNP and -[Mn^{III}]@CSNP, respectively.

	Samples			
	0%	0.9%	1.2%	4.2%
	BET			
V_t (cm^3/g) [a]	0.48	0.45	0.34	0.26
$a_{S,BET}$ (m^2/g) [b]	313	332	274	187
C_{BET}	900	468	434	284
	t-plot			
a_{int} (m^2/g) [c]	206	228	196	158
a_{ext} (m^2/g) [d]	39	38	35	35
V_{micro} (cm^3/g) [c]	4.7×10^{-2}	4.4×10^{-2}	3.4×10^{-2}	1.3×10^{-2}
V_{meso} (cm^3/g) [d]	0.19	0.21	0.17	0.11
V_{meso}/V_t	0.39	0.46	0.50	0.42
	BJH			
D_{BJH} (nm) [e]	2.4	2.4	2.7	2.4
D_{min} (nm) [f]	1.4	-	-	-
D_{max} (nm) [g]	4.8	4.8	5.4	5.4

From BET plot (a) for $p/p_0 = 0.99$; (b) $0.05 \geq p/p_0 \geq 0.16$. Calculated from t-plot (c) for $t \leq 0.5$; (d) for $t \geq 1.0$. From BJH plot, with $D_i = 2r_i$ (e) for $\frac{dV_p}{dr_p}_{\max}$; (f) for $\frac{dV_p}{dr_p}_{\max} - \left(\frac{dV_p}{dr_p}_{\max}\right)/2$; (g) for $\frac{dV_p}{dr_p}_{\max} + \left(\frac{dV_p}{dr_p}_{\max}\right)/2$.

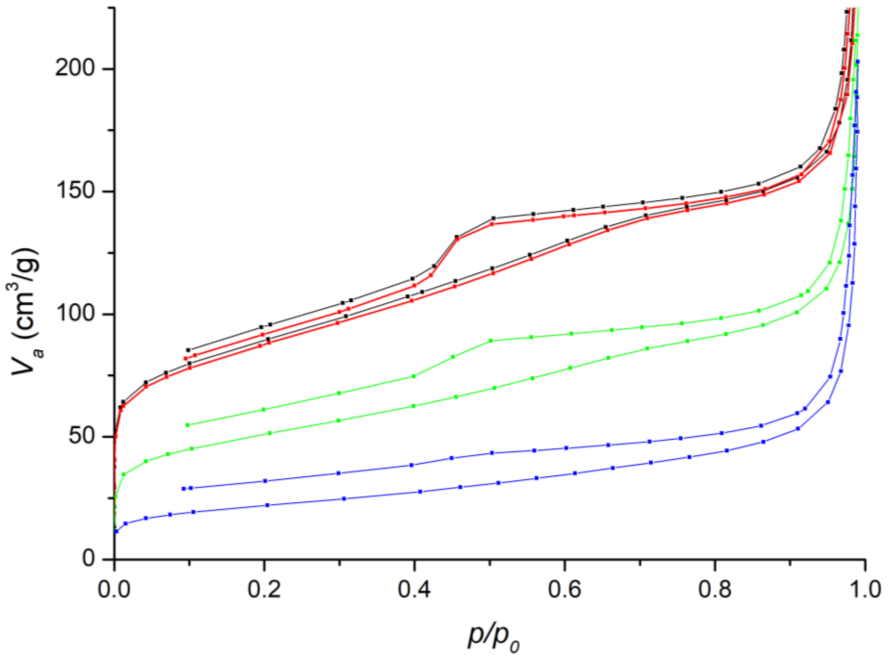

Figure S17. N_2-sorption isotherms at 77 K of [Mn^{II}]@CSNP loaded with 50 (blue), 30 (green) and 10 mg (red) of Mn^{II} complex for 1.00 g of CSNPs, 5-[Mn^{II}]@CSNP, 3-[Mn^{II}]@CSNP and 1-[Mn^{II}]@CSNP, respectively. Black line and points are the isotherms of the complex-free CSNP.

Table S7. Porosity data from N_2-sorption isotherms for [Mn^{II}]@CSNP: BET: Total pore volume (V_t), BET area ($a_{S,BET}$) and C coefficient (C_{BET}); t-plot : internal (a_{int}) and external (a_{ext}) pore area and microporous (V_{meso}) and mesoporous (V_{meso}) volume; microporous ratio (V_{micro}/V_t) of hybrid carbon-silica nanoparticles. The head-column number for each sample is the [Mn^{II}] complex load deduced from material Mn elemental analysis, which corresponds to CSNP, 1-[Mn^{II}]@CSNP, 3-[Mn^{II}]@CSNP and -[Mn^{II}]@CSNP, respectively.

	Samples			
	0%	0.4%	2.9%	4.6%
	BET			
V_t (cm^3/g) [a]	0.48	0.43	0.33	0.29
$a_{S,BET}$ (m^2/g) [b]	313	307	174	74
C_{BET}	900	576	330	197
	t-plot			
a_{int} (m^2/g) [c]	206	210	133	61
a_{ext} (m^2/g [d])	39	37	37	36
V_{micro} (cm^3/g) [c]	4.7×10^{-2}	4.3×10^{-2}	1.8×10^{-2}	5.6×10^{-3}
V_{meso} (cm^3/g) [d]	0.19	0.19	0.10	3.0×10^{-2}
V_{meso}/V_t	0.39	0.43	0.30	0.10
	BJH			
D_{BJH} (nm) [e]	2.4	2.4	2.4	2.4
D_{min} (nm) [f]	1.4	1.4	1.4	1.4
D_{max} (nm) [g]	4.8	4.8	5.4	5.4

From BET plot (a) for $p/p_0 = 0.99$; (b) 0.05$\geq p/p_0 \geq$0.16. Calculated from t-plot (c) for $t \leq 0.5$; (d) for $t \geq 1.0$. From BJH plot, with $D_i = 2r_i$ (e) for $\frac{dV_p}{dr_p}_{\max}$; (f) for $\frac{dV_p}{dr_p}_{\max} - \left(\frac{dV_p}{dr_p}_{\max}\right)/2$; (g) for $\frac{dV_p}{dr_p}_{\max} + \left(\frac{dV_p}{dr_p}_{\max}\right)/2$.

Magnetic Properties

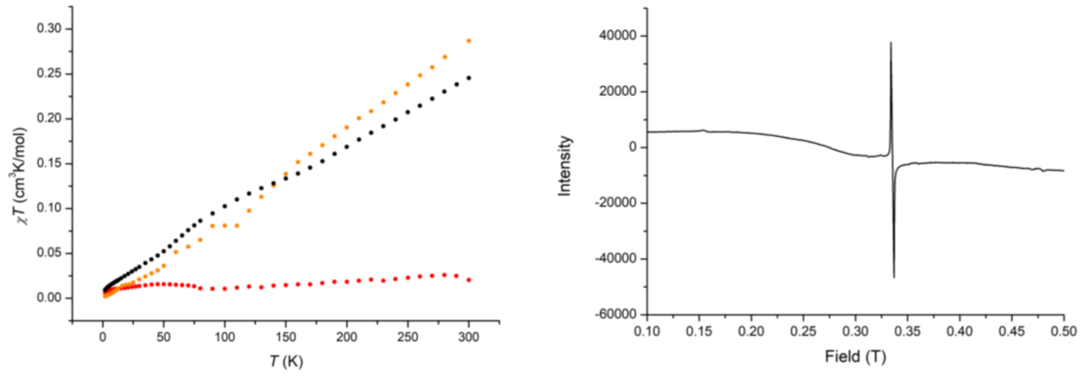

(**a**) (**b**)

Figure S18. (**a**) Molar magnetic susceptibility times temperature plotted versus temperature for RSNP support (red circles), SNP support (orange circles) and CSNP support (black circles); (**b**) X-band EPR spectrum of CSNP (powder) recorded at 12K.

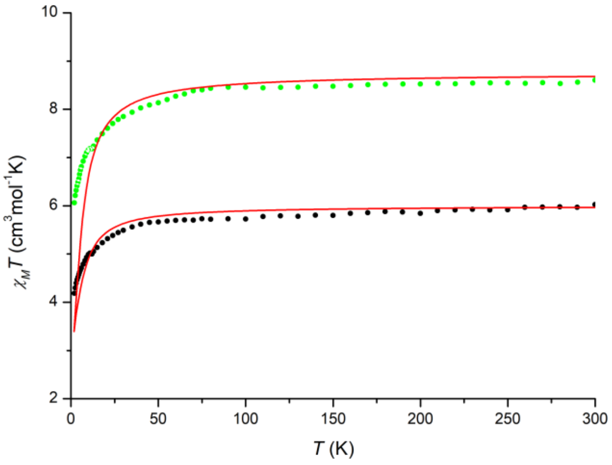

Figure S19. Molar magnetic sensibility times temperature plotted versus temperature for [Mn^{II}]@RSNP (green circles) and [Mn^{III}]@RSNP (black circles). The simulated curves for each material are represented in red.

Luminescent Properties

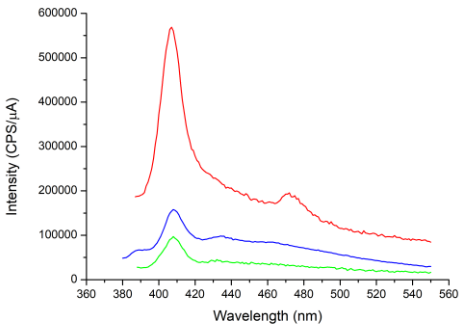

(**a**)

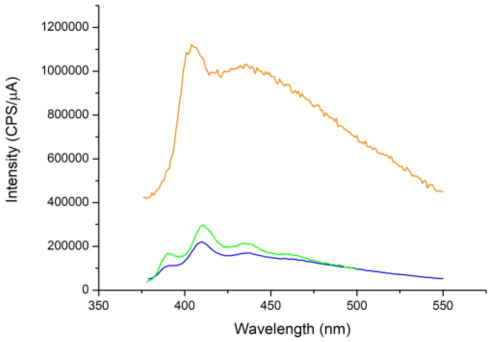

(**b**)

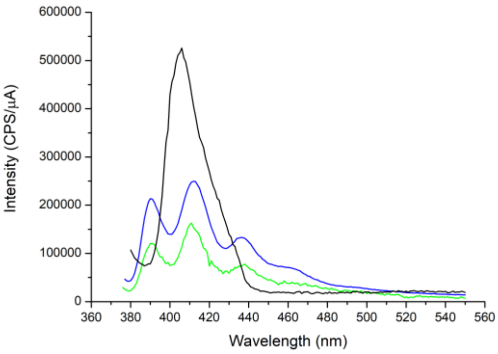

(**c**)

Figure S20. Emission spectra of [Mn]@NP with the smallest Mn compound load for each NP type compared to the emission spectra of empty NP. Suspensions correspond all to 250 mg/L of NP in ethanol. Excitation wavelength: 362 nm. Concentrations refer to Mn compounds. (a) RSNP (red), $[Mn^{II}]^{0.6}$@RSNP (green) (1.1×10^{-7} mol/L), $[Mn^{III}]^{1.0}$@RSNP (1.1×10^{-7} mol/L) (blue) (b) SNPs (orange), $[Mn^{II}]^{1.2}$@SNP (2.3×10^{-7} mol/L) (green), $[Mn^{III}]^{0.9}$@SNP (1.0×10^{-7} mol/L) (blue) and (c) CSNP (black), $[Mn^{II}]^{0.4}$@CSNPs (6.7×10^{-8} mol/L) (green) and $[Mn^{III}]^{0.9}$@CSNP (1.0×10^{-7} mol/L) (blue).

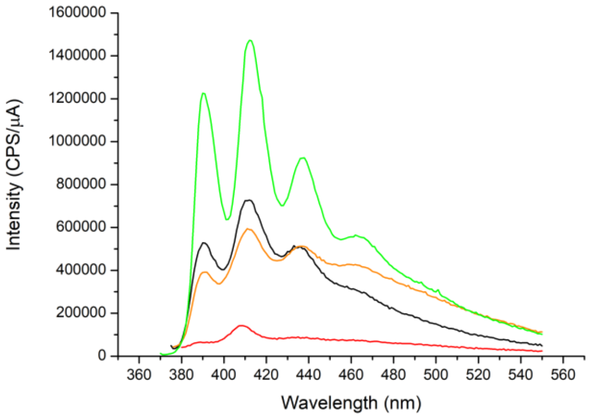

Figure S21. Emission spectra of free [Mn^{II}] (green line), [Mn^{II}]@RSNP (red line), [Mn^{II}]@SNP (orange line and [Mn^{II}] CSNP (black line). Concentration of [Mn^{II}] is 5.5 × 10^{-7} mol/L in ethanol for each solution/suspension. Excitation wavelength: 362 nm.

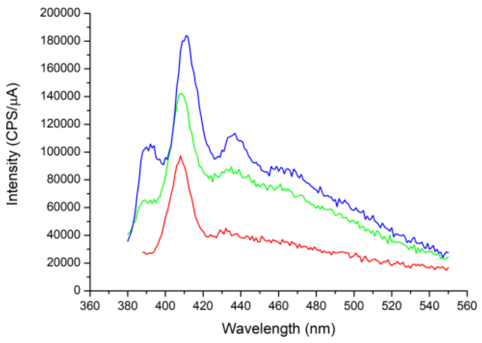

(**a**)

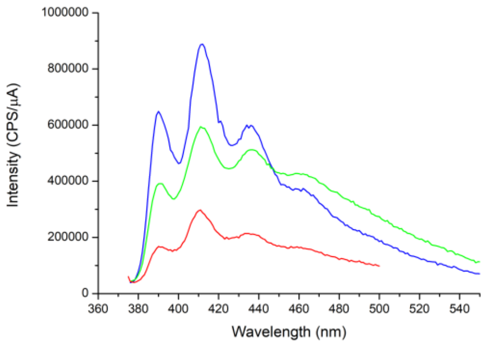

(**b**)

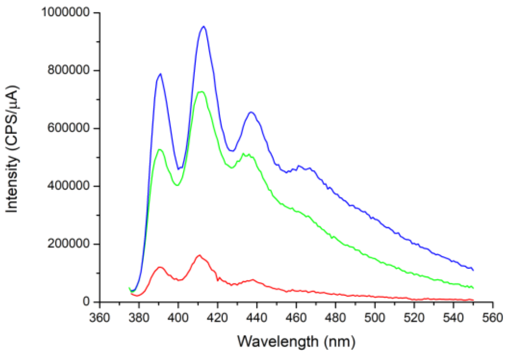

(**c**)

Figure S22. Emission spectra of [Mn^{II}]@NP for each NP type. For each graph presented, the first value is the [Mn^{II}] load of the particle in wt%, the second the concentration of [Mn^{II}] is the suspension in mol/L. (a) (on the upper left) RSNP: 0.6 wt%; 1.1 × 10^{-7} mol/L (red line); 2.0 wt%; 3.9 × 10^{-7} mol/L (green line) ; 2.9 wt% ; 5.5 × 10^{-7} mol/L (blue line). (b) (on the upper right) SNP: 1.2 wt%; 2.3 × 10^{-7} mol/L (red line) ; 2.9 wt% ; 5.5 x 10^{-7} mol/L (green line) ; 3.9 wt% ; 7.3 × 10^{-7} mol/L (blue line). (c) (in the lower middle) CSNP: 0.4 wt%; 6.7 × 10^{-8} mol/L (red line) ; 2.9 wt% ; 5.5 × 10^{-7} mol/L (green line) ; 4.6 wt% ; 8.7 × 10^{-7} mol/L (blue line).

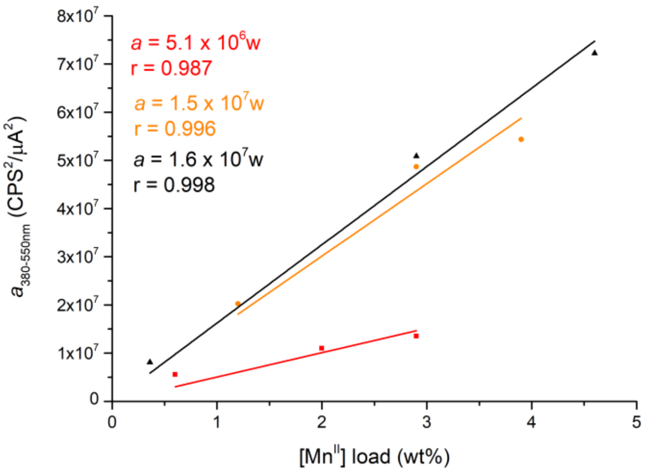

Figure S23. Area below the emission curve versus particle's load for [Mn^{II}] of [Mn^{II}]@RSNP (red squares), [Mn^{II}]@SNP (orange circles) and [Mn^{II}]@CSNP (black triangles) suspensions. Linear fits with their equations are plotted with matching colours.

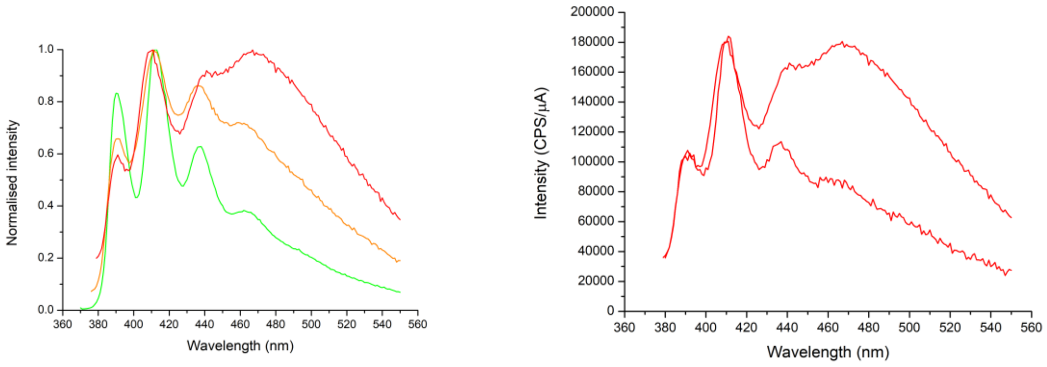

(**a**) (**b**)

Figure S24. (**a**) Normalised emission spectra of [Mn^{II}]$^{2.9}$@RSNP (5.5×10^{-7} mol/L) (red line), [Mn^{II}]$^{2.9}$@SNP (5.5×10^{-7} mol/L) (orange line) and free [Mn^{II}] (5.5×10^{-7} mol/L) (green line) in ethanol. (**b**) Emission spectra of two [Mn^{II}]$^{2.9}$@RSNP (5.5×10^{-7} mol/L) suspensions in ethanol.

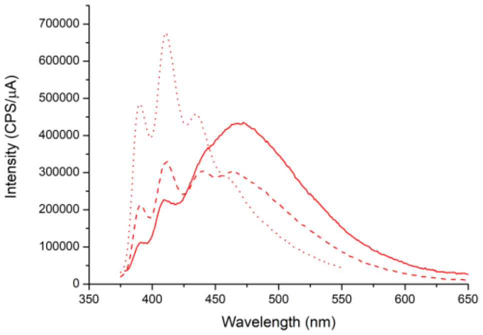

(**a**)

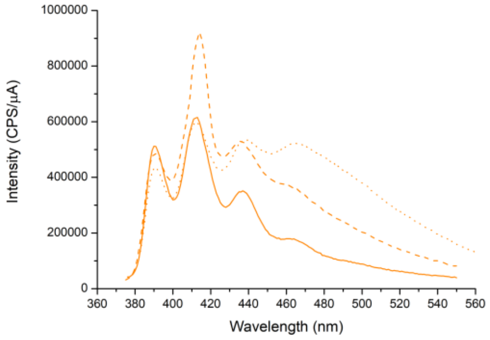

(**b**)

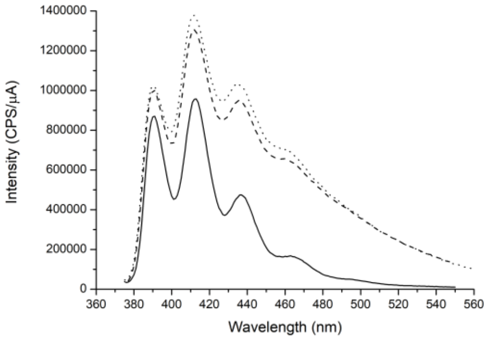

(c)

Figure S25. Emission spectra of [Mn^{III}]@NP for each NP type (full line) compared to the emission spectra of the supernatant after centrifuagtion (dashed line) and the filtrate (dotted line). (**a**) [Mn^{III}]$^{4.6}$@RSNP (5.5 × 10^{-7} mol/L) (**b**) [Mn^{III}]$^{3.8}$@SNP (4.6 × 10^{-7} mol/L) and (**c**) [Mn^{III}]$^{4.2}$@CSNP (5.0 × 10^{-7} mol/L).

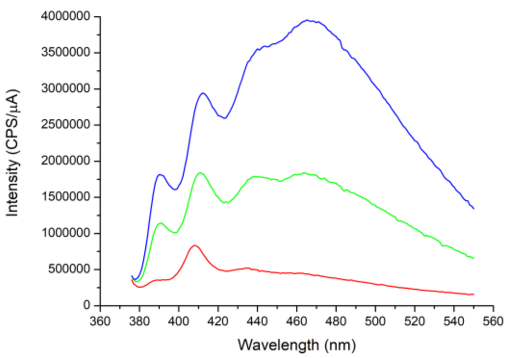

(**a**)

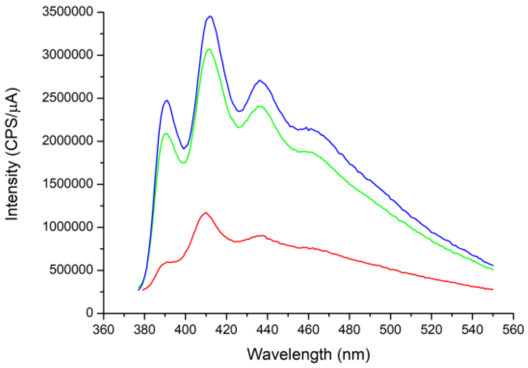

(**b**)

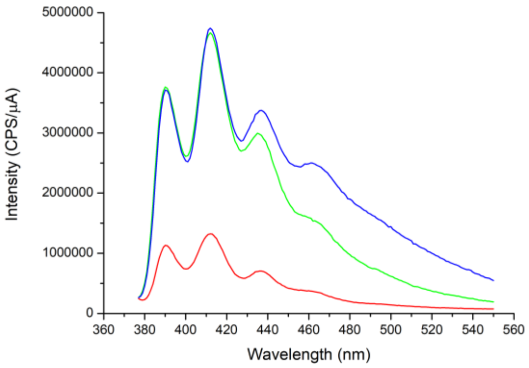

(c)

Figure S26. Emission spectra of [Mn^{III}]@NP for each NP type. For each graph presented, the first value is the [Mn^{III}] load of the particle in wt%, the second the concentration of [Mn^{III}] is the suspension in mol/L. (**a**) (on the upper left) RSNP: 1.0 wt% ; 1.1 × 10^{-7} mol/L (red line) ; 2.7 wt% ; 3.2 × 10^{-7} mol/L (green line) ; 4.6 wt% ; 5.5 × 10^{-7} mol/L (blue line). (**b**) (on the upper right) SNP: 0.9 wt%; 1.0 × 10^{-7} mol/L (red line); 2.5 wt%; 2.9 × 10^{-7} mol/L (green line) ; 3.8 wt% ; 4.6 × 10^{-7} mol/L (blue line). (**c**) (in the lower middle) CSNP: 0.9 wt%; 1.0 × 10^{-7} mol/L (red line) ; 1.2 wt% ; 1.5 × 10^{-7} mol/L (green line) ; 4.2 wt% ; 5.0 × 10^{-7} mol/L (blue line).

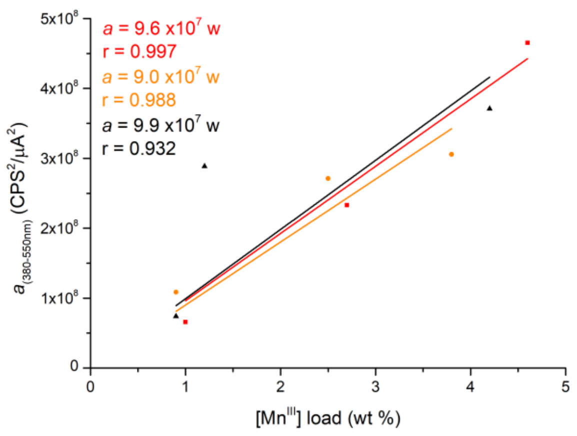

Figure S27. Area below the emission curve versus particle's load for [Mn^{III}] of [Mn^{III}]@RSNP (red squares), [Mn^{III}]@SNP (orange circles) and [Mn^{III}]@CSNP (black triangles) suspensions. Linear fits with their equations are plotted with matching colours.
